# Allostery in the dengue virus NS3 helicase: Insights into the NTPase cycle from molecular simulations

**DOI:** 10.1371/journal.pcbi.1006103

**Published:** 2018-04-16

**Authors:** Russell B. Davidson, Josie Hendrix, Brian J. Geiss, Martin McCullagh

**Affiliations:** 1 Department of Chemistry, Colorado State University, Fort Collins, Colorado, United States of America; 2 Department of Microbiology, Immunology, and Pathology, Colorado State University, Fort Collins, Colorado, United States of America; 3 School of Biomedical Engineering, Colorado State University, Fort Collins, Colorado, United States of America; Bogazici University, TURKEY

## Abstract

The C-terminus domain of non-structural 3 (NS3) protein of the *Flaviviridae* viruses (e.g. HCV, dengue, West Nile, Zika) is a nucleotide triphosphatase (NTPase) -dependent superfamily 2 (SF2) helicase that unwinds double-stranded RNA while translocating along the nucleic polymer. Due to these functions, NS3 is an important target for antiviral development yet the biophysics of this enzyme are poorly understood. Microsecond-long molecular dynamic simulations of the dengue NS3 helicase domain are reported from which allosteric effects of RNA and NTPase substrates are observed. The presence of a bound single-stranded RNA catalytically enhances the phosphate hydrolysis reaction by affecting the dynamics and positioning of waters within the hydrolysis active site. Coupled with results from the simulations, electronic structure calculations of the reaction are used to quantify this enhancement to be a 150-fold increase, in qualitative agreement with the experimental enhancement factor of 10–100. Additionally, protein-RNA interactions exhibit NTPase substrate-induced allostery, where the presence of a nucleotide (e.g. ATP or ADP) structurally perturbs residues in direct contact with the phosphodiester backbone of the RNA. Residue-residue network analyses highlight pathways of short ranged interactions that connect the two active sites. These analyses identify motif V as a highly connected region of protein structure through which energy released from either active site is hypothesized to move, thereby inducing the observed allosteric effects. These results lay the foundation for the design of novel allosteric inhibitors of NS3.

## Author summary

Non-structural protein 3 (NS3) is a *Flaviviridae* (e.g. Hepatitis C, dengue, and Zika viruses) helicase that unwinds double stranded RNA while translocating along the nucleic polymer during viral genome replication. As a member of superfamily 2 (SF2) helicases, NS3 utilizes the free energy of nucleotide triphosphate (NTP) binding, hydrolysis, and product unbinding to perform its functions. While much is known about SF2 helicases, the pathways and mechanisms through which free energy is transduced between the NTP hydrolysis active site and RNA binding cleft remains elusive. Here we present a multiscale computational study to characterize the allosteric effects induced by the RNA and NTPase substrates (ATP, ADP, and P_i_) as well as the pathways of short-range, residue-residue interactions that connect the two active sites. Results from this body of molecular dynamics simulations and electronic structure calculations are highlighted in context to the NTPase enzymatic cycle, allowing for development of testable hypotheses for validation of these simulations. Our insights, therefore, provide novel details about the biophysics of NS3 and guide the next generation of experimental studies.

## Introduction

Flaviviruses (family *Flaviviridae*) are small (∼11 kilobases) positive-sense, single-stranded RNA (ssRNA) viruses that include members such as dengue (serotypes 1-4), Zika, West Nile, yellow fever, and Japanese Encephalitis viruses. The dengue virus (DENV) is a public health threat that causes serious morbidity and mortality globally [1, 2]. Infection with DENV can result in “break-bone” fever, an extraordinarily painful disease with symptoms ranging from a mild fever to a fatal hemorrhagic syndrome [3]. There are approximately 50 million serious infections and 20,000 deaths each year, and dengue infections are a leading cause of mortality in children in a number of Latin and Asian countries [1]. Dengue viruses have re-emerged in the United States, and a growing number of locally acquired infections in Florida, Texas, and Hawaii have been reported over the last decade. Despite a reinvigorated effort due to the recent Zika epidemic [4], there are currently no approved small molecule antivirals to treat Flavivirus-induced diseases.

One of the primary antiviral targets in *Flaviviridae* is the nonstructural protein 3 (NS3), which plays a critical role in the viral replication cycle [5–15]. NS3 is a multifunctional protein found in all *Flaviviridae*, possessing an N-terminal serine protease domain responsible for proteolytically cleaving the viral polyprotein during translation [16] and a C-terminal helicase/nucleotide triphosphatase (NTPase)/RNA triphosphatase domain [17–22]. In a nucleotide triphosphate (NTP) hydrolysis-dependent mechanism, the NS3 helicase domain (NS3h) unwinds double-stranded RNA (dsRNA) while translocating along the nucleic polymer. These functions are required to resolve the dsRNA replication intermediate into fully-mature positive strand RNAs (see Ref. [23] for a recent review). Mutations in the NS3 helicase and NTPase active sites are seen to abrogate NS3 function as well as decrease viral survival [24–26], demonstrating the importance of these enzymatic functions to the flavivirus life cycle. Drugs identified to inhibit DENV NS3h suffer from specificity issues because they are either NTPase inhibitors [27] or RNA/DNA mimics such as ivermectin [13], suramin [14] or aurintricarboxylic acid [15]. Therefore, it is of interest to further elucidate the mechanism of DENV NS3h with molecular resolution to help identify new and specific target regions for antiviral therapeutics.

**Fig 1:**
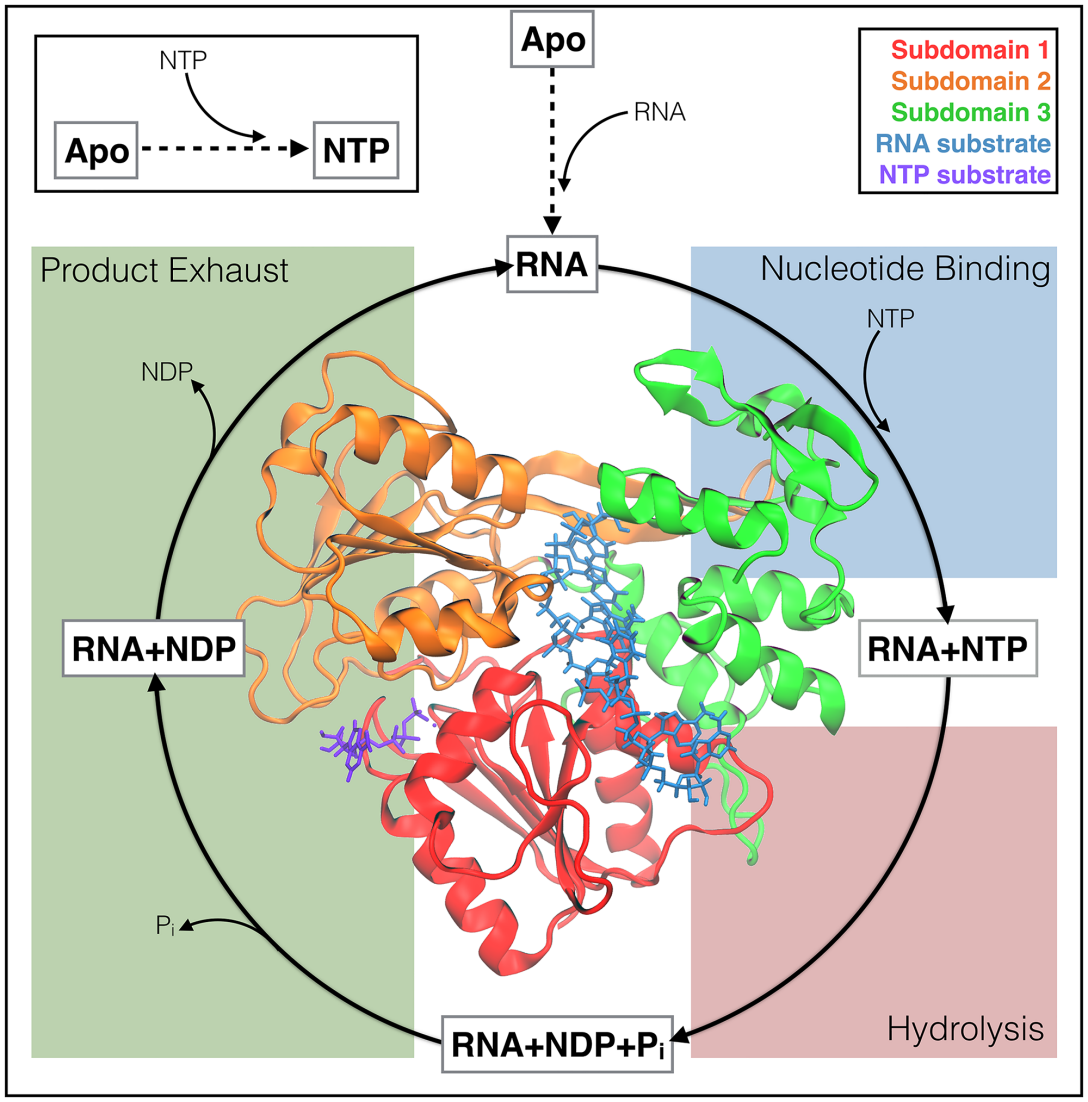
The NTPase cycle of NS3h. A schematic depicting the hypothesized substrate cycle that NS3h moves through during the NTPase function. Free energy released from this cycle powers the unwinding of dsRNA and unidirectional translocation along the nucleic polymer. The protein structure (inset) demonstrates the tertiary structure of NS3h as well as the positions of the RNA-binding cleft (ssRNA substrate colored blue) and the NTPase active site (ATP molecule colored purple).

The *Flaviviridae* NS3h have been classified as a superfamily 2 (SF2) helicase (NS3/NPH-II subfamily; a DEx/H helicase) where the NTPase cycle (Fig 1) provides the free energy needed to unwind dsRNA and translocate along the nucleic substrate in a 3′ to 5′ direction [28]. Structurally, NS3h are monomeric helicases composed of three subdomains; subdomains 1 and 2 (red and orange in the inset of Fig 1) are RecA-like folds that are structurally conserved across all SF1 and SF2 helicases, whereas subdomain 3 (green) is unique to the NS3/NPH-II subfamily and contains some of the least conserved portions of the protein. In Fig 1, an adenosine triphosphate (ATP; purple) molecule is bound within the NTPase active site between subdomains 1 and 2. Also, an RNA substrate (blue) is bound within the RNA-binding cleft, separating subdomains 1 and 2 from subdomain 3. The 5′ terminus of the RNA is positioned at the top of the protein in Fig 1 and the ds/ss RNA junction is hypothesized to be just above this region of the protein.

The NS3/NPH-II subfamily of SF2 helicases exhibit both RNA-stimulated NTPase activity and NTPase-dependent helicase activity [17–22]. These experimentally observed phenomena suggest that (1) the presence of RNA affects the NTPase active site, thereby activating the NTPase cycle and (2) this cycle is the source of free energy needed to perform work on the RNA (translocation and unwinding). In Fig 1, the enzymatic cycle for the NTPase function is depicted by four dynamic events: RNA is bound within the RNA-binding cleft and activates the NTPase cycle, NTP binds, NTP is hydrolyzed, and finally products (nucleotide diphosphate—NDP—and inorganic phosphate—H_2_PO_4_^-^, P_i_) are released. To date, it is unclear which stage(s) of the cycle are responsible for the translocation and unwinding functions of NS3h. Furthermore, the biophysical couplings between NTPase and helicase active sites are still poorly understood [28].

One of the better studied *Flaviviridae* NS3h is that of the Hepatitis C virus (HCV; family: *Flaviviridae hepacivirus*) [29–40]. Utilizing both ensemble [29–35] and single molecule [36–38, 41, 42] techniques, studies have provided insights into the kinetic steps of the HCV NS3h translocation function. These studies, alongside crystallography studies of various *Flaviviridae* NS3h, suggest that the NS3 enzyme tracks along the phosphodiester backbone of the nucleic oligomer, unwinding one base-pair per hydrolysis event [35–37]. To explain these experimental results, various models describing the translocation mechanism have been reported, depicting NS3h as a Brownian [33–35] or backbone stepping motor [36, 39–41] protein. These models envision the coupling between NTPase and helicase functions through different biophysical mechanisms, yet the models are not mutually exclusive and are limited in temporal and spatial resolution [43, 44].

Luo *et al*. reported a set of crystal structures of the DENV NS3h in important protein-substrate complexes of the NTPase cycle (bolded text in Fig 1) [45]. From these structures, major allosteric influences of RNA-binding were seen in the NTPase active site. For example, Luo and coworkers noted that the presence of an RNA substrate shifts the carboxylate group of Glu285 (motif II) into a more catalytically relevant structure for the hydrolysis reaction. Mutation of the Glu285 residue abrogates NTPase and helicase activities [25]. These static structures have provided novel insights into RNA-induced protein structural changes yet provide limited insight into the NTPase cycle or translocation and unwinding functions of NS3h.

Previous theoretical studies of helicases have focused on a broad range of enzymes such as PcrA (SF1) [46–49], transcription terminator Rho (SF5) [50], SV40 (SF3) [51], and various NS3h enzymes [52–56]. Of the theoretical studies on NS3h, Perez-Villa *et al*. reported microsecond-long molecular dynamics (MD) simulations of the HCV NS3h-ssRNA systems in the presence and absence of ATP and ADP. The reported simulations were used to interrogate the thermodynamics of these substrate states with various conformations of the NTPase active site [52]. While the reported results are of interest for NS3h, the authors provide limited insight into the molecular mechanisms at play during the NTPase cycle. Other theoretical studies of the NS3h enzyme are limited in timescales (tens to hundreds of ns of simulation), substrate states modeled, or spatial resolution (e.g. coarse grained elastic network model) [53–56]. Therefore, theoretical modeling of the NS3h enzyme has yet to elucidate further details about the structural and dynamic couplings within NS3h in light of the NTPase cycle.

We report here a multiscale theoretical study of the DENV NS3h enzyme at each substrate state along the NTPase cycle. RNA-induced allostery on the NTPase active site is reported wherein the presence of an RNA substrate alters the positioning and dynamics of waters within the hydrolysis active site. Inspired by this observation, minimum energy electronic structure calculations are performed to investigate the energy landscape of the hydrolysis reaction. Additionally, investigations into NTPase substrate-induced allostery on the RNA-binding cleft suggest that NS3h interacts with RNA in a NTPase substrate-dependent manner. Umbrella sampling (US) simulations are performed to enhance the sampling of a proposed elementary step of the translocation mechanism observed during the unbiased simulations. Finally, analyses of the correlated motions between residues are used to identify allosteric pathways that connect the two active sites. It is through these pathways that we hypothesize that free energy released during the NTPase cycle is transduced to the RNA-binding cleft and utilized to perform work on the RNA. This study of the substrate states of DENV NS3h lays the foundation for further study of the NTPase cycle and marks the most complete picture of the molecular mechanism of the NS3 NTPase/helicase to date.

## Methods and models

### Starting structures and system preparation

A subset of the crystal structures reported by Luo *et al*. [45] of the Dengue NS3h (serotype 4) are used as the initial structures for all-atom, explicit solvent MD simulations. Specifically, the binary complex of NS3h with a seven-residue ssRNA substrate (PDB ID: 2JLU) is used to model the ssRNA substrate state, while the ternary structures of ssRNA+ATP (2JLV), ssRNA+ADP+P_*i*_ (2JLY), and ssRNA+ADP (2JLZ) model the pre-hydrolysis, post-hydrolysis, and product release states of the NTPase cycle, respectively. The Apo (2JLQ) and ATP (2JLR) substrate states are also simulated and used as experimental controls for our investigation into allostery.

The RNA-bound structures of DENV NS3h were crystalized as dimers of the protein [45]. For these systems, chain A of the structure is used as the starting conformation. Furthermore, the A conformers are chosen for residues with multiple side chain conformations. In all crystal structures with ATP substrates, the crystalized Mn^2+^ divalent cation is converted into a Mg^2+^. For the ATP crystal structure (2JLR), residues of the protease linker region were poorly resolved and so are transferred from the Apo (2JLQ) structure after aligning the neighboring amino acid backbones in both systems.

### Molecular dynamics simulations

All-atom, explicit solvent MD simulations are performed for the six substrate states of DENV NS3 and presented in Fig 1 (denoted Apo, ATP, ssRNA, ssRNA+ATP, ssRNA+ADP+P_i_, and ssRNA+ADP). The simulations are performed using the GPU-enabled AMBER14 software [57], ff14SB [58] parameters for proteins, and ff99bsc0_*χ* OL3_ [59, 60] parameters for RNA. Parameters for ATP [61], ADP [61], P_i_ (provided in Supplementary Information (SI); S2 File), and Mg^2+^ [62] are also used. For each system, the crystal structures are solvated in TIP3P water boxes with at least a 12 Å buffer between the protein and periodic images. Crystallographic waters are maintained. Sodium and chloride ions are added to neutralize charge and maintain a 0.10 M ionic concentration. The Langevin dynamics thermostat and Monte Carlo barostat are used to maintain the systems at 300 K and 1 bar. Direct nonbonding interactions are calculated up to a 12 Å distance cutoff. The SHAKE algorithm is used to constrain covalent bonds that include hydrogen [63]. The particle-mesh Ewald method [64] is used to account for long-ranged electrostatic interactions. A 2 fs integration time step is used, with energies and positions written every 2 ps. The minimum amount of simulation performed for each system is one trajectory of 1.5 *μ*s, with the first 200 ns of simulation sacrificed to equilibration of the starting structures. Simulation of the ssRNA system is performed to 2 *μ*s. For both the ATP and ssRNA+ATP systems, two 1.5 *μ*s simulations are performed. The total amount of unbiased simulation reported here on the described structures is 12.5 *μ*s.

### Umbrella sampling simulations

US simulations are performed to enhance sampling of a hypothesized elementary translocation event wherein the biased collective variable is the distance between the central carbon of the guanidinium group of Arg387 to the phosphorous atom of phosphate 4 in the RNA. These simulations are run for the ssRNA, ssRNA+ATP, ssRNA+ADP+P_i_, and ssRNA+ADP systems, using the same protocol as the unbiased simulations with the addition of a bias. For each substrate state, a minimum of 22 sampling windows are simulated for 50 ns each with harmonic wells positioned every 0.5 Å and ranging from 3.50 to 14.00 Å. Harmonic force constants are 20 kcal mol^-1^ Å^-2^. Further simulation and additional windows are run in regions of collective variable space with poor sampling. The weighted histogram analysis method (WHAM) [65] is used to analyze the results of these simulations, with bin sizes of 0.1 Å. Bootstrapping is used to approximate error bars for the probability density and free energy plots shown. The total amount of biased simulations reported here is 5.12 *μ*s.

### Electronic structure calculations

Electronic structure calculations are performed at the *ω*B97X-D/6-31+G* level of theory [66] using the Guassian 09 version B.01 program [67]. The *ω*B97X-D functional is chosen due to its broad applicability [68, 69] and a recent study demonstrating its energetic accuracy for a variety of phosphate hydrolysis reactions [70]. The QM system is composed of a truncated ATP molecule (truncated to methyl triphosphate, MTP), functional groups of nine surrounding protein residues (Pro195, Gly196, Lys199, Glu285, Ala316, Gly414, Gln456, Arg460, and Arg463), a Mg^2+^ ion, and seven water molecules. The amino acids are truncated at various positions (more detail in S1 File) using hydrogen atoms. For each residue, the position of the terminal heavy atom is frozen to maintain the active site geometry. This yielded a total of 138 atoms in the QM calculations.

These calculations are performed on active site conformations pulled from the unbiased MD simulations of the ssRNA+ATP and ATP substrate states, thereby investigating the influence of observed RNA structural allostery on the hydrolysis reaction mechanism and energy landscape. Frames used for the initial reactant state structures were selected by visualizing MD frames in which a lytic water is present. Through visual and RMSD analyses of such frames, a single frame was chosen to represent the population of catalytically relevant structures. The hydrolysis reaction is then monitored by optimizing the reactants (MTP+lytic water), products ($\text{MDP}+{\text{HPO}}_{4}^{2-}$), and a single transition state (TS) in between. The initial TS and product state structures were created from the previous optimized structure. The minima are confirmed using a Hessian calculation. The TS is confirmed by examining the direction of the single imaginary frequency. Following geometry optimization, frequency calculations are performed to obtain gas-phase, zero-point energy corrected free energies for each active site conformation.

### Data analysis

Unless stated otherwise, analyses of MD trajectories are performed using Python 2.7 and the MDAnalysis module (version 0.15.0) [71]. Matplotlib is used for plotting data [72]. VMD is used for visualization of trajectories and production of structural figures [73–75]. For each substrate state, a single frame from the trajectories is used when presenting structural details of the respective substrate state. Further information on choosing these “exemplar” structures is given in the S1 File. Additionally, details of all analyses performed can be found in the S1 File. All scripts for the analyses are available on Github (https://github.com/mccullaghlab/DENV-NS3h).

## Results and discussion

For clarity, we present and discuss our results in three sections. The first and second sections independently report observed RNA-induced and NTPase substrate-induced structural allosteries, respectively. The focus of the RNA-induced allostery section is on the structural changes seen in the NTPase active site due to bound RNA. Similarly, the NTPase substrate-induced allostery section highlights changes seen in the structure and dynamics of the RNA-binding cleft due to the presence of different nucleotide substrates. In the final section, correlated motions between residues are used to highlight pathways through which these structural allosteric effects are induced.

### RNA-induced allostery

To date, no biophysical explanation has been proposed for the 10 to 100-fold increase in NTPase turnover rate observed for DENV NS3h in the presence of RNA [22]. Crystallographic studies of the DENV NS3h structure have identified static structural allostery due to RNA binding [45], yet a dynamic picture and interpretation of these influences are still missing. In this section, comparisons of the simulations of the Apo, ATP, ssRNA, and ssRNA+ATP substrate states are used to depict structural rearrangements induced by RNA. These RNA-induced allosteries are observed to affect the positioning and dynamics of waters within the NTPase active site. These novel insights gained from the comparisons of the MD simulations inspire the reported electronic structure calculations of the reactant, transition, and product states of the hydrolysis reaction. In combination, these results demonstrate that the observed enhancement of NTPase activity originates from the RNA-induced destabilization of the lytic water.

#### The RNA-binding loop and *α*2

**Fig 2:**
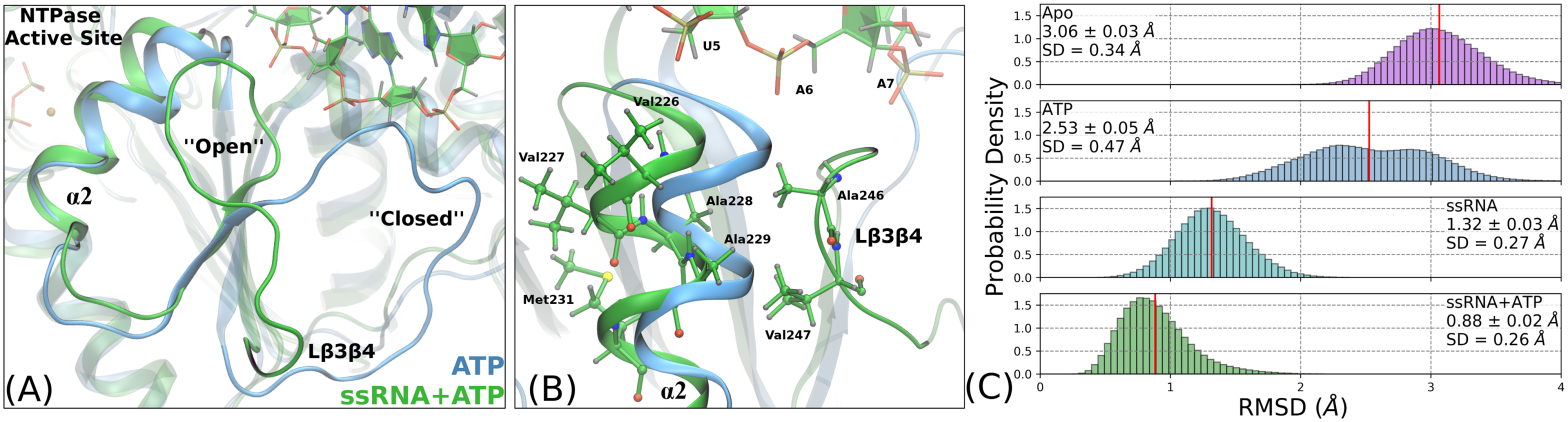
RNA-induced displacement of L*β*3*β*4 and *α*2. (A) Depiction of the “open” and “closed” structural states of L*β*3*β*4 for exemplar structures of ATP (blue) and ssRNA+ATP (green) simulations. (B) Hydrophobic interactions between L*β*3*β*4 and *α*2 stabilize the “open” conformation. Furthermore, Val227 and Met231 (*α*2) are pushed in towards the NTPase active site when L*β*3*β*4 is in the “open” conformation. (C) RMSD of *α*2 (residues 224 to 235) backbone atoms referenced against the ssRNA+ATP crystal structure (PDB ID: 2JLV).

The most marked structural difference between DENV NS3h with and without ssRNA is the change in conformation of the RNA-binding loop (L*β*3*β*4; Thr244 to Glu255). The crystal structures of DENV NS3h from Luo *et al*. [45] with no RNA present (Apo, 2JLQ; ATP, 2JLR) resolve this loop in a “closed” conformation while the crystal structures with bound RNA all have this loop in an “open” conformation. Fig 2(A) depicts both conformations and the relative position of the loop with respect to the RNA-binding cleft and NTPase active site. In the “closed” conformation, the RNA-binding loop is covering part of the RNA-binding cleft while, in the “open” conformation, this loop contacts the phosphodiester backbone of the RNA as well as amino acids of *α*-helix 2 (*α*2). Transitions from “closed” to “open” conformations are not sampled during our MD simulations of the Apo and ATP systems demonstrating that the crystal structure conformations are minima in the solution phase free energy surfaces.

The RNA-induced structural change of L*β*3*β*4 affects the position of *α*2 as highlighted in Fig 2(B), where the top of *α*2 is displaced in towards the NTPase active site when L*β*3*β*4 is in the “open” conformation. This conformation is stabilized by hydrophobic contacts between Ala246 and Val247 (L*β*3*β*4) and Val226, Ala228, Ala229 (*α*2). When in the “closed” conformation, this hydrophobic pocket is not formed and leaves the top of *α*2 exposed to solvent.

The structural deviation of *α*2 is quantified by computing the root mean square deviation (RMSD) of the backbone atoms of *α*2 (residues 224 to 235) relative to the ssRNA+ATP crystal structure (2JLV). The distributions of this metric are presented in Fig 2(C) with the largest structural deviations seen in the simulations of the Apo and ATP substrate states. Bound RNA decreases the RMSD values while an ATP substrate shows minimal influence. Therefore, these RNA-induced hydrophobic interactions between L*β*3*β*4 and *α*2 stabilize the structural conformation of *α*2 where the top of the helix is pushed in towards the NTPase active site. Interestingly, Val227 and Met231 are the residues in *α*2 that have prominent positions in the NTPase active site. While these hydrophobic side chains likely have minimal influence on the hydrolysis reaction mechanism, their structural shift into the hydrolysis active site reduces the volume of the pocket.

#### Motif II

Motif II (Walker B) is a set of highly conserved amino acid residues within NTPase enzymes and is known to play an important role in the catalysis of the hydrolysis reaction [25]. In DENV and other *Flaviviridae*, motif II is the DEAH sequence (residues 284 to 287) where Asp284 and Glu285 are positioned in the rear of the NTPase active site. Luo *et al*. noted that the presence of RNA shifts the carboxylate group of Glu285 from a magnesium-bound position to a position more conducive to coordinating the lytic water [45]. In this RNA-induced position, Glu285 is ideally located to act as a base where it can accept a proton from the lytic water, thereby increasing the nucleophilicity of the attacking group during the hydrolysis reaction.

**Fig 3:**
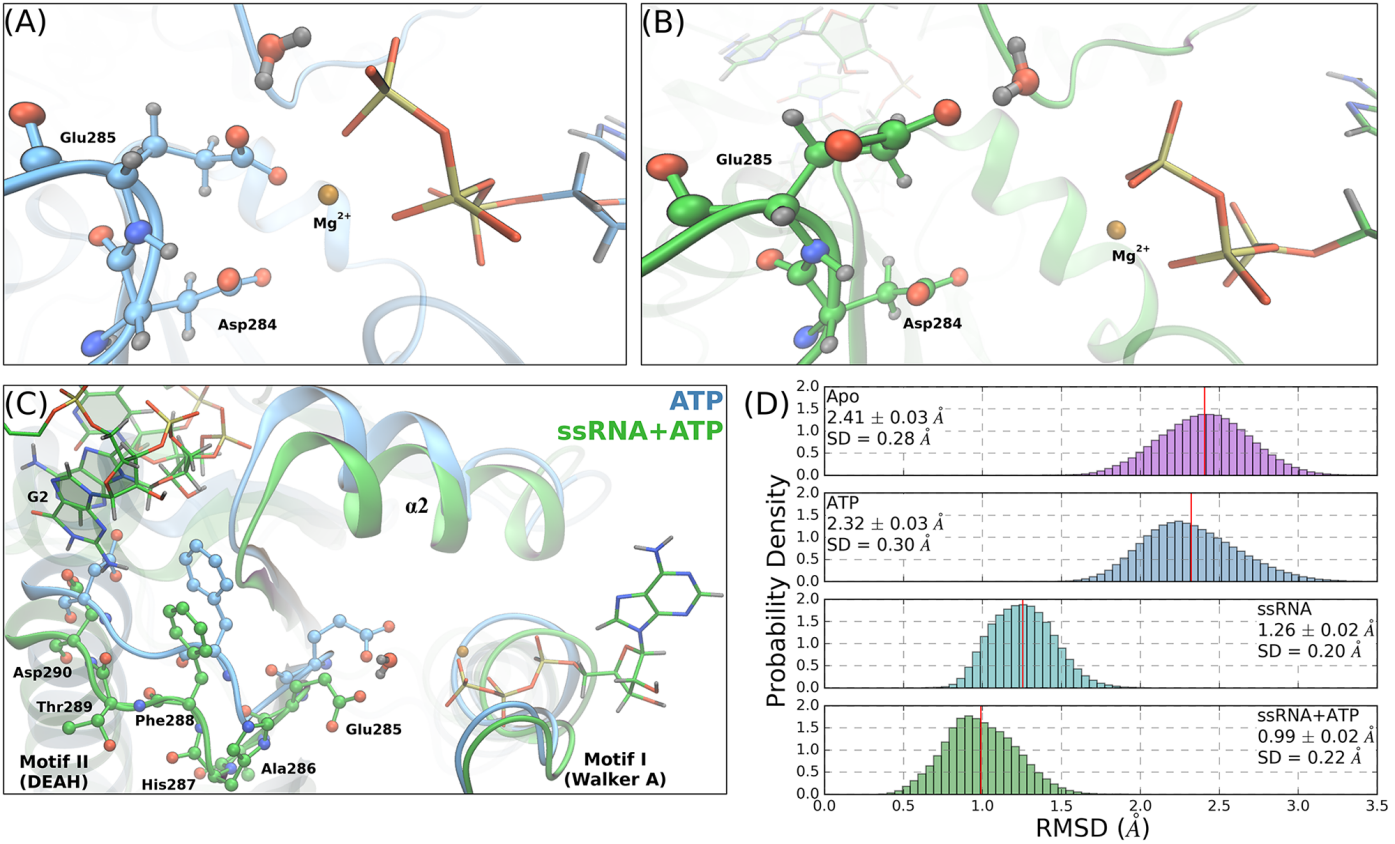
RNA-induced allostery on motif II. The Asp284 and Glu285 positioning relative to the *γ*-phosphate of the ATP molecule, for the ATP (A) and ssRNA+ATP (B) systems. In each panel, the highlighted water molecule is identified as the most lytic-like water within the active site. (C) Structural alignment of the same frames shown in (A) and (B), highlighting the RNA-induced backbone shift of residues Glu285 to Asp290. Phe288 and Asp290 are highlighted in both systems due to their prominence in the RNA-binding cleft. (D) RMSD of the backbone atoms of residues 284 to 290 referenced against the ssRNA+ATP crystal structure (PDB ID: 2JLV).

Our MD simulations maintain these starting conformations and support the deduced importance of Glu285. Snapshots of the Glu285 positions in the ATP and ssRNA+ATP simulations are shown in Fig 3(A) and 3(B), respectively. The highlighted water demonstrates the position of a lytic water in the NTPase active site, relative to the *γ*-phosphorus atom. Structurally, with RNA bound, the carboxylate side chain of Glu285 is pulled away from the coordination sphere of the Mg^2+^ cation and is moved into plane with the terminal phosphoanhydride bond. In either position, Glu285 is observed to hydrogen bond with the lytic water yet, in the RNA-induced position, the lytic water is positioned in a more ideal environment for nucleophilic attack (quantified in the next section).

Both Asp284 and Glu285 are major structural landmarks within the NTPase active site and have no direct interactions with the RNA substrate. Rather, the origin of the RNA-induced structural rearrangement of motif II residues is attributed to RNA-induced displacement of residues down the linear amino acid sequence, specifically Phe288 and Asp290. Fig 3(C) shows the structural alignment of the ATP and ssRNA+ATP structures (same frames as in panels (A) and (B)), focusing on residues Glu285 to Asp290. The structural deviations of the residues highlighted in Fig 3(C) are quantified with an RMSD analysis of the backbone atoms of residues 284 to 290, referenced against the ssRNA+ATP crystal structure (Fig 3(D)). There is a shift of ∼ 1.3 Å in these atoms when comparing RNA-bound systems (ssRNA, ssRNA+ATP) and no RNA systems (Apo, ATP). Therefore, bound RNA causes a backbone shift of the post-motif II residues (e.g. Phe288, Asp290) that propagates to the residues within the NTPase active site.

#### Water positioning and dynamics within the NTPase active site

RNA allosterically affects the positions of amino acids within the NTPase active site, yet it is unclear how these structural rearrangements influence the hydrolysis cycle. When comparing the ATP and ssRNA+ATP simulations, the positions and dynamics of the ATP molecule and Mg^2+^ cation are minimally affected by the presence of RNA. Alternatively, waters within the NTPase active site are observed to be greatly influenced by the presence of bound RNA. For example, the average number of water molecules found within the NTPase active site decreases from 30.0±0.7 molecules for the Apo substrate state to 21.72±0.08 molecules in the ssRNA state. A similar but reduced trend is observed when comparing the ATP (15.0±0.2 water molecules) and ssRNA+ATP (12.8±0.4) simulations.

**Fig 4:**
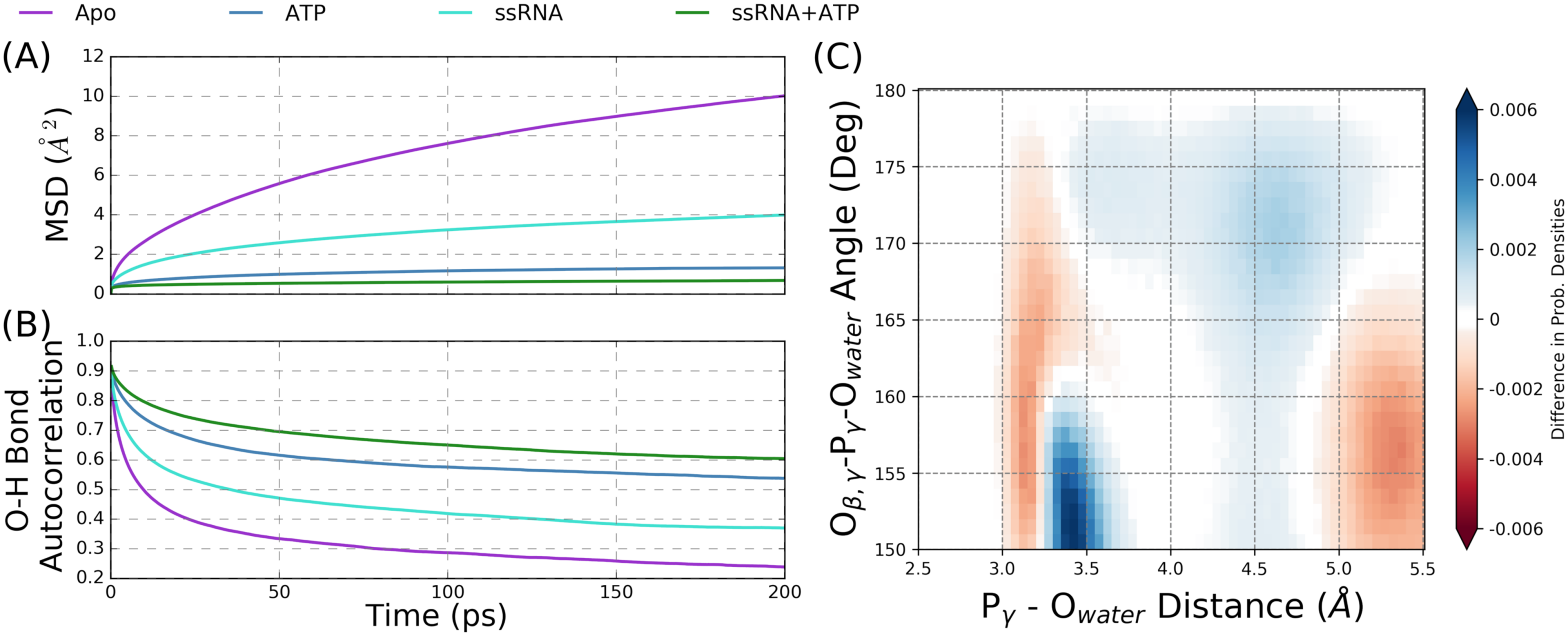
Water dynamics and positioning within the NTPase active site. (A) Mean square displacement (MSD) and (B) O-H bond autocorrelation metrics for the Apo, ATP, ssRNA, and ssRNA+ATP simulations that describe the translational and rotational motions of waters within the active site. (C) The difference between the ssRNA+ATP and ATP probability densities of water positions within the NTPase active site, projected onto the O_*β*,*γ*_-P_*γ*_-O_wat_ angle and P_*γ*_-O_wat_ distance. These axes are used to project water positions into catalytically relevant space relative to the ideal position of a lytic water in the hydrolysis reaction.

The translational and rotational dynamics of water molecules within the NTPase active site are also influenced by bound RNA, as shown graphically in Fig 4(A) and 4(B). The mean squared displacement (MSD, panel (A)) is a metric describing the average squared distance traveled by water molecules within the NTPase active site over a time interval, where large slopes indicate fast diffusion of water. The MSD metric for the Apo substrate state (purple) has a large slope relative to the ssRNA substrate state, demonstrating that waters in the NTPase active site diffuse more slowly when an RNA is bound within the binding cleft. Although much less dramatic, a similar trend is seen in the ATP and ssRNA+ATP states. The O-H bond autocorrelation metric (panel (B)) describes the rotational motions of water molecules within the active site, thereby looking at water reorientation; slower decay of this metric indicates slower reorientation times. Similar to the MSD results, the ssRNA-bound systems have extended O-H bond correlation times relative to the control states (Apo and ATP), indicating that rotational motions of water molecules within the NTPase active site are slowed by the RNA.

Considering the hypothesized SN2 mechanism of the hydrolysis reaction [76, 77], ideal nucleophilic attack by a lytic water on the *γ*-phosphorous atom (P_*γ*_) of ATP is described by an attack angle of 180° with respect to the terminal phosphoanhydride (P_*γ*_-O_*β*,*γ*_) bond. The distance between the lytic water oxygen (O_wat_) and P_*γ*_ will decrease to a bonded distance of ∼ 1.7 Å over the course of this reaction. Therefore, the P_*γ*_-O_wat_ distance and O_*β*,*γ*_-P_*γ*_-O_wat_ angle are used as geometric collective variables that describe the nucleophilic attack of a lytic water. Projecting the positions of waters within the NTPase active site onto these two coordinates allows for comparisons of the positioning of catalytically relevant water in the ATP and ssRNA+ATP substrate simulations. The two-dimensional heat maps of this projection are shown in the SI (Figs G and H in S1 Text) for both of the substrate states.

The difference between the probability densities for the ssRNA+ATP and ATP simulations is shown in Fig 4(C), where positive values (blue) correspond to increased probability density in ssRNA+ATP versus ATP states. Therefore, the presence of RNA causes water molecules in lytic positions of the NTPase active site to shift into more ideal (larger) nucleophilic angles while pushing competing waters at short distances to lower angles. Motivated by the electronic structure calculations reported in the next section, geometric cutoffs are used to quantify these observations by defining a conical volume of the NTPase active site within which waters are identified as lytic: waters with a P_*γ*_-O_wat_ distance less than 5.0 Å and an O_*β*,*γ*_-P_*γ*_-O_wat_ angle greater than 155° are defined as lytic. The probability of observing a frame with water in a lytic position is 72.93% ± 0.04% for the ATP system and 79.08% ± 0.03% for the ssRNA+ATP system.

In total, these results demonstrate that RNA affects the dynamics and positioning of waters within the NTPase active site. These effects are propagated from the RNA binding cleft to the NTPase active site through structural rearrangements of L*β*3*β*4, *α*2, and motif II. Although it is difficult to fully deconvolute the specific influences of these structural allosteries on the water molecules in the active site, we propose that the observed influence of RNA on number and dynamics of water molecules originates from the structural rearrangement of *α*2, where Val227 and Met231 become more prominent in the hydrolysis active site when RNA is bound. These hydrophobic residues not only exclude water molecules from the active site but also slow the translational and rotational motions of water molecules. This RNA-induced effect can be thought of as a entropic destabilization of the NTPase active site, where the RNA decreases the phase space that the water molecules can populate. Furthermore, the RNA-induced structural rearrangement of the Glu285 carboxylate group leads to the observed increased in probability of lytic water molecules. Through the backbone displacement of motif II residues, the Glu285 side chain is pulled away from the Mg^2+^ cation and into plane of the *γ*-phosphate group, thereby creating a local protein environment that stabilizes water molecules into more ideal positions for nucleophilic attack. This effect is interpreted as a direct destabilization of the lytic water in the hydrolysis reaction.

#### Electronic study of the NTP hydrolysis reaction

**Fig 5:**
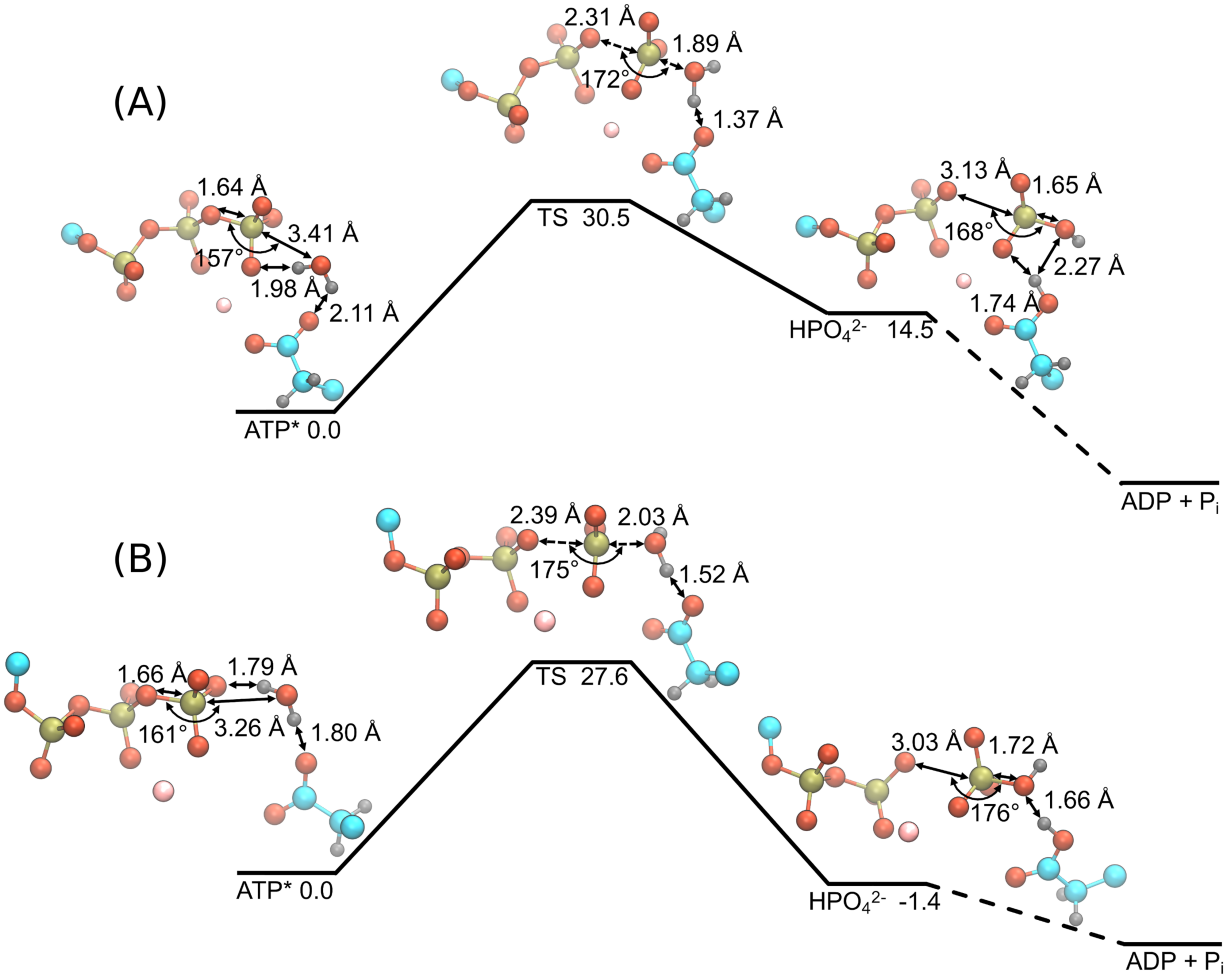
Energy landscape and structures of the NTP hydrolysis reaction in the active site of DENV NS3h for the ATP (A) and ssRNA+ATP (B) substrate states. DFT calculations were performed using the *ω*B97X-D/6-31+G* level of theory. A total of 138 atoms were included in the quantum mechanical calculations (see supporting information for full structures) but only the triphosphate, lytic water, Mg^2+^, and Glu285 side chain atoms are shown here for clarity. Important distances and angles are included in the structural representations of each state. All energies are reported in units of kcal mol^−1^.

The impact of the RNA-induced repositioning of the lytic water on the hydrolysis reaction is investigated using density functional theory (DFT) calculations of an abbreviated NTPase active site where conformations are pulled from the unbiased MD simulations. Active site geometry optimizations are performed on the ATP and ssRNA+ATP substrate states where the hydrolysis reaction is modeled as a concerted SN2 mechanism using a reactant state (ATP*), a transition state (TS), and a product state (${\text{HPO}}_{4}^{2-}$). Geometry optimized potential energies and gas phase free energy corrections are used to compute the free energy landscape of the hydrolysis reaction for the respective substrate state, as presented in Fig 5. Figure A in the S1 Text highlights the full selection of the NTPase active site (amounting to 138 atoms) that are included in the DFT calculations. For clarity, the geometries presented in Fig 5 only include the triphosphate, lytic water, Mg^2+^, and Glu285 atoms.

The free energy landscape of the ATP substrate state is presented in Fig 5(A), where the reactant structure has the lytic water 3.41 Å away from the gamma phosphorus and at an angle of 157° between the water oxygen and the O_*β*,*γ*_-P_*γ*_ bond. The TS structure is found to be 30.5 kcal mol^−1^ above the reactant state, with a P_*γ*_-O_wat_ distance of 1.89 Å and O_*β*,*γ*_-P_*γ*_-O_wat_ angle of 172°. Additionally, the O_*β*,*γ*_-P_*γ*_ bond distance has increased from 1.64 Å to 2.31 Å. Over the transition, the lytic water molecule reorients relative to the *γ*-phosphate and Glu285 atoms. Via this reorientation, a proton from the lytic water is partially transferred to the carboxylate group of Glu285, as seen in the difference in the water O-H bond distance between TS and reactant states (ΔO_wat_ − H_wat_ = d(O_wat_ − H_wat_)_TS_ − d(O_wat_ − H_wat_)_ATP*_ = 0.12 Å). The product state was found following the TS in which the proton has completely transferred to Glu285, forming the HPO_4_^2-^ molecule.

Panel (B) of Fig 5 depicts the hydrolysis reaction landscape and structures for the ssRNA+ATP substrate state. The reactant structure has the lytic water 3.26 Å away from the gamma phosphorus and at an O_*β*,*γ*_-P_*γ*_-O_wat_ angle of 161°. In the TS structure, the P_*γ*_-O_wat_ and O_*β*,*γ*_-P_*γ*_ distances become 2.03 Å and 2.39 Å, respectively, while the O_*β*,*γ*_-P_*γ*_-O_wat_ angle increases to 175°. Unlike in the ATP substrate state, the O-H bond distance of the lytic water is minimally perturbed when comparing the reactant and TS structures (ΔO_wat_-H_wat_ = 0.04 Å). Rather, the proton transfer step is completed during the transition from the TS to the product state. Overall, the calculated activation barrier height for the ssRNA+ATP substrate state is 27.6 kcal mol^−1^, corresponding to a 2.8 kcal mol^−1^ decrease in barrier height relative to the ATP substrate state landscape.

The overall reaction, $\text{ATP}\left(aq\right)\overset{\text{NS3h}}{\to }\text{ADP}\left(aq\right)+{\text{P}}_{i}\left(aq\right)$, is expected to be exergonic for both substrate states due to NS3h being hydrolysis active in the presence and absence of RNA [22]. While the energy landscape for the ATP substrate state (panel (A)) does not demonstrate an exergonic reaction, we hypothesize that neither product states presented in Fig 5 adequately model the final product state of the hydrolysis reaction. This hypothesized thermodynamic product state requires a proton transfer from Glu285 to the ${\text{HPO}}_{4}^{2-}$ molecule as well as an unbinding event of the ${\text{HPO}}_{4}^{2-}$ molecule from the Mg^2+^ coordination sphere. Optimization of such a product state is infeasible due to the limited description of the protein environment in these DFT calculations. Additionally, it is assumed that the energy barriers of these subsequent events are much smaller than the hydrolysis reaction barrier and so, are disregarded in the current study.

The calculated activation barrier heights of both substrate states are in good agreement with previous DFT studies of NTP hydrolysis in a protein environment [77–83] and in aqueous solution [84–86]. The differences observed between the ATP and ssRNA+ATP free energy landscapes of the hydrolysis reaction are mainly attributed to the different positions of the Glu285 carboxylate group. For either substrate state, this functional group acts as a base that increases the nucleophilicity of the lytic water. When unbound from the Mg^2+^ coordination sphere, Glu285 performs this function more effectively, stabilizing the lytic water at a shorter P_*γ*_-O_wat_ distance and a larger O_*β*,*γ*_-P_*γ*_-O_wat_ angle. Additionally, comparisons of the associative (P_*γ*_-O_wat_ distance) and dissociative (O_*β*,*γ*_-P_*γ*_ distance) reaction coordinates suggest that the RNA-induced structural rearrangement of the active site leads to slight changes in the hydrolysis mechanism. For both substrate states, the change in P_*γ*_-O_wat_ distance from reactant to TS states (ATP: ΔP_*γ*_-O_wat_ = -1.52 Å; ssRNA+ATP: ΔP_*γ*_-O_wat_ = -1.23 Å) is larger in magnitude than the respective change in O_*β*,*γ*_-P_*γ*_ distance (ATP: ΔO_*β*,*γ*_-P_*γ*_ = 0.67 Å; ssRNA+ATP: ΔO_*β*,*γ*_-P_*γ*_ = 0.73 Å). These values suggest that the hydrolysis reaction proceeds through an asynchronous SN2 hydrolysis mechanism where the nucleophilic attack of the lytic water is enhanced by the local protein environment [87, 88]. Further comparison of these values demonstrates that the hydrolysis reaction for the ATP substrate state proceeds through a more “associative” [88] transition state than does the reaction for the ssRNA+ATP substrate state. The changes in the reaction coordinates discussed above as well as the changes in the O_wat_-H_wat_ distance (ATP: ΔO_wat_-H_wat_ = 0.12 Å; ssRNA+ATP: ΔO_wat_-H_wat_ = 0.04 Å) and the O_Glu285_-H_wat_ distance (ATP: ΔO_Glu285_-H_wat_ = -0.74 Å; ssRNA+ATP: ΔO_Glu285_-H_wat_ = -0.28 Å) collectively suggest that the ssRNA+ATP TS structure is more reactant-like than the TS structure of the ATP substrate state.

#### Theoretical enhancement factor of the RNA-stimulated NTPase activity

The results from the last two subsections are consistent with the hypothesis that the biophysical origin of the experimentally observed RNA-stimulated NTPase activity [22] is two-fold: (1) the RNA-induced structural changes of L*β*3*β*4, *α*2, and motif II affect the probability of water molecules to be located in lytic positions, and (2) the same RNA-induced structural changes alter the activation barrier of the hydrolysis reaction. To account for both effects, a reaction scheme is proposed to describe the NTPase function within NS3h, where a fast equilibrium exists between active site conformations with and without the presence of a lytic water. This equilibrium is followed by the slow, irreversible hydrolysis reaction. Using this scheme, the theoretical observed rate constant for the NTP hydrolysis reaction is k_obs_ = K_eq_k_hydrol_. For both ATP and ssRNA+ATP substrate states, the K_eq_ is defined as the respective ratio of probabilities of observing a MD frame with and without a lytic water. The hydrolysis rate constant is quantified using an Arrhenius rate equation where the Boltzmann factor accounts for the activation energy barrier observed in the electronic structure calculations. The ratio of observed rate constants is defined as the theoretical enhancement factor of the RNA-stimulated NTPase activity,

$$\begin{array}{c} \text{Enhancement}\;\text{Factor}=\frac{k_{obs}^{ssRNA+ATP}}{k_{obs}^{ATP}}=\frac{K_{eq}^{ssRNA+ATP}}{K_{eq}^{ATP}}\exp(-\Delta E_{a}/RT) \end{array}$$ (1)

where Δ*E*_*a*_ is the difference in activation energies between the ssRNA+ATP and ATP substrate states ($E_{a}^{ssRNA+ATP}-E_{a}^{ATP}$). The Arrhenius prefactor is assumed to be constant for both ATP and ssRNA+ATP substrate states and thus does not contribute to the enhancement factor.

Taking the ratio of the ssRNA+ATP and ATP rates results in a theoretical enhancement factor of 150, which is consistent with the experimentally observed enhancement factor (10 to 100) [22]. Error analyses for this calculation are at least three fold: (1) statistical error from the sampling in MD simulations, (2) error in the force field, and (3) error in the DFT calculations. Propagation of the statistical uncertainties of the probability of observing a lytic water lead to an error in the enhancement factor of 0.002. The errors in the force field and the DFT energies [89] are difficult if not impossible to estimate. Uncertainty of the enhancement factor is comprised mainly of errors in the activation energies obtained from the DFT calculations since they are present in the exponential term in the Arrhenius equation. Therefore, an error of 1 kcal mol^−1^ in the activation barriers is used as a conservative estimate for the DFT method. Using these approximated uncertainties, the minimum and maximum values for the calculated enhancement factor are observed to bound the best estimate (150) by an order of magnitude on both sides. Even with this large range, the calculated enhancement factor maintains the qualitative narrative that RNA-induced structural allostery of L*β*3*β*4, *α*2, and motif II leads to the repositioning of the lytic water within the NTPase active site as well as affects the energetics of the hydrolysis reaction.

### NTPase substrate-induced allostery

Experimental studies have shown that the NS3h helicase functions (translocation and unwinding) are NTPase dependent, yet it is unclear which equilibrium states and/or dynamic events of the NTPase cycle are the source of the necessary free energy for these functions [20, 21]. All previously developed models describing these functions have deduced that the NTPase cycle drives conformational changes in the RNA-binding cleft, thereby cycling the protein-RNA interactions leading to unidirectional translocation and melting of the duplex/single stranded nucleic junction [33–36, 39–41]. Yet, limited structural allostery attributed to the NTPase substrates (e.g. ATP, ADP, and P_i_) is observed in the crystal structures of DENV NS3h [45]. Therefore, a subset of the MD simulations reported here (ssRNA, ssRNA+ATP, ssRNA+ADP+P_i_, and ssRNA+ADP) is used to interrogate protein-RNA interactions as well as identify protein structural changes that have NTPase substrate-dependent behaviors.

#### Protein-RNA contacts

**Fig 6:**
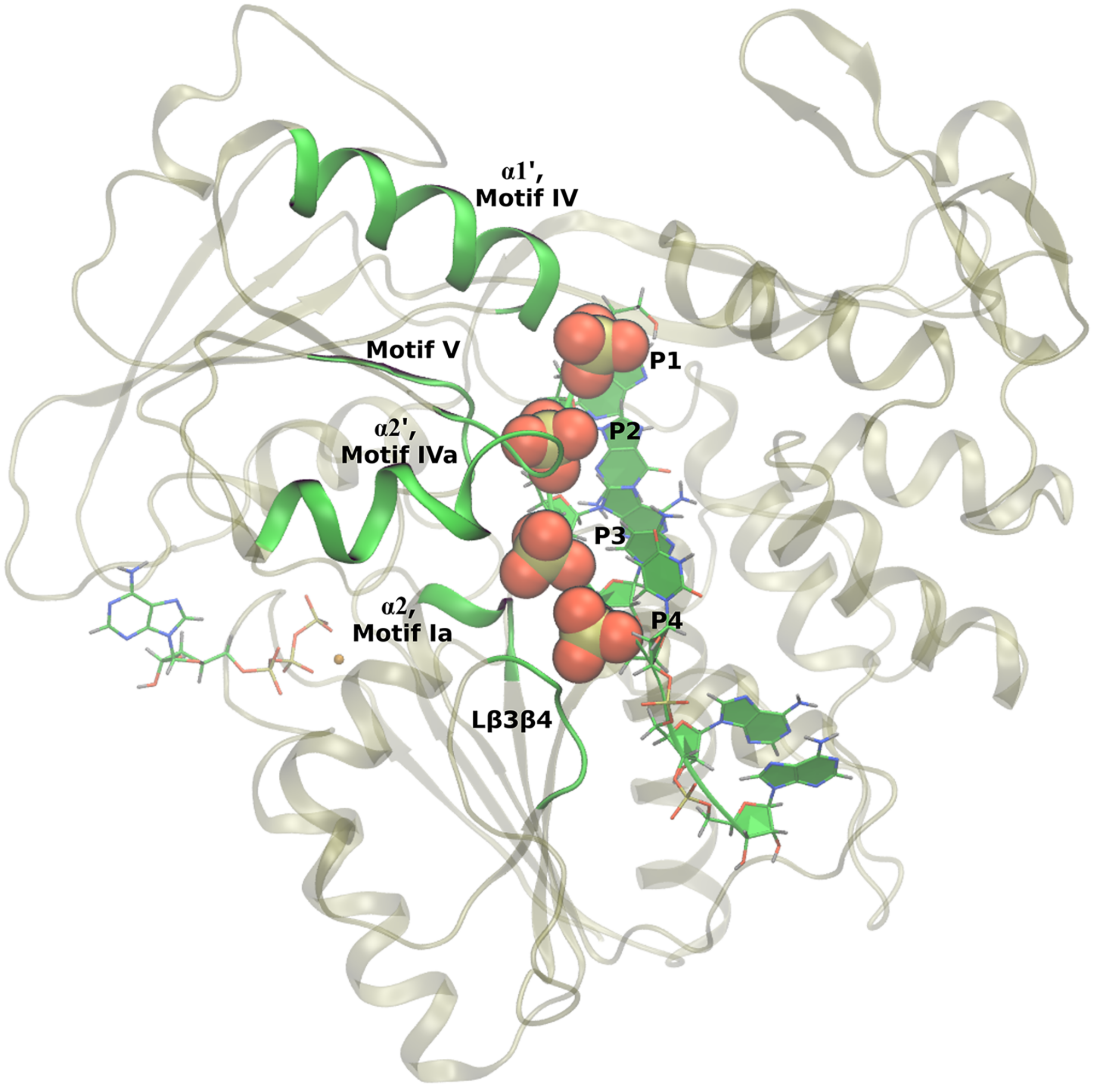
Protein-RNA contacts. Motifs Ia, IV, IVa, V as well as L*β*3*β*4 of NS3h make strong contact with the phosphodiester backbone of the RNA. These contacts are dominated by electrostatic interactions between the phosphate groups of RNA and highly conserved amino acid residues. The four strongly bound phosphate groups (labeled P1 through P4) are highlighted with space filling representations.

For the RNA-bound substrate states modeled here, the RNA oligomer is seven residues long with the first five residues (5′ end) strongly bound within the RNA-binding cleft of the protein. The remaining two nucleic residues are poorly resolved in the crystal structures [45] and are highly fluctional in the MD simulations. For the NS3/NPH-II helicase subfamily, amino acids in motifs Ia, IV, IVa, and V as well as L*β*3*β*4 are observed in crystal structures to have contact with the phosphodiester backbone of the nucleic oligomer [39, 45], yet little is known about the dynamic role that these residues play during translocation and unwinding of the nucleic substrate [28]. As observed in our MD simulations, protein-RNA contacts are dominated by electrostatic interactions between highly conserved residues in these motifs and the first four phosphate groups of the ssRNA, as highlighted in Fig 6. Specifically, arginines (225 and 387), threonines (224, 244, and 408), backbone amides (Arg225, Ile365, Arg387), and *α*-helix dipole moments (*α*2 as well as subdomain 2 *α*-helices 1, *α*1′, and 2, *α*2′) [90] are observed to stabilize RNA through interactions with the phosphate groups.

#### Asymmetry of the protein-RNA interactions

Nonbonding interaction energies are used to provide a quantitative description of the relative strength of the protein-RNA interactions. Comparisons of these data between substrate states of the NTPase cycle provide insight into the hypothesized NTPase dependence of protein-RNA interactions. The pair-wise sum of Lennard-Jones and short-range, unscreened electrostatic energies are calculated using the *lie* analysis function in AMBER14 cpptraj [91]. Table 1 shows the nonbonding interaction energies between RNA phosphates 1 through 4 (P1-4) (in total and individually) and all protein residues. An interaction cutoff of 12 Å was used for these calculations.

**Table 1:** Nonbonding interaction energies between RNA phosphate groups (named P1 through P4) and all protein residues.

	ssRNA	ssRNA+ATP	ssRNA+ADP+P_i_	ssRNA+ADP
**P1-4**	-582 ± 6	-625 ± 3	-649 ± 5	-579 ± 4
**P1**	-62 ± 2	-106 ± 2	-126 ± 4	-118 ± 3
**P2**	-157 ± 2	-192 ± 1	-193 ± 1.3	-158 ± 1.9
**P3**	-206 ± 3	-188.4 ± 0.4	-190.2 ± 0.9	-176.9 ± 0.5
**P4**	-155 ± 3	-139.3 ± 0.6	-139.7 ± 0.9	-126 ± 1.3

Units for all values shown are kcal mol^-1^. An interaction cutoff of 12 Å is used. Short-range, electrostatic energies were calculated with a dielectric of 1.

The totals of interaction energies between the protein and P1-4 demonstrate that the ssRNA+ATP (-625 ± 3 kcal mol^-1^) and ssRNA+ADP+P_i_ (-649 ± 5 kcal mol^-1^) structures have more stable protein-RNA interactions than the ssRNA (-582 ± 6 kcal mol^-1^) and ssRNA+ADP (-579 ± 4 kcal mol^-1^) structures. These results suggest that the presence of the *γ*-phosphate group (or P_i_) in the NTPase active site has a stabilizing effect on the strongly bound phosphate groups of the RNA. This result is in direct disagreement with experimental results of HCV NS3 where it is observed that the protein has a decrease in affinity for nucleic oligomers in the presence of ATP [32, 34]. One possible reason for this discrepancy is the lack of a realistic nucleic polymer that extends above and below the RNA-binding cleft in the simulations reported here. Modeling more complex RNA structures is left for further study.

The interaction energies between protein atoms and individual phosphate groups presented in Table 1 allow for comparisons of local protein-RNA interactions in the various substrate states. Protein-phosphate 2 (P2) energies show similar trends as the total energies, where the presence of the *γ*-phosphate group stabilizes P2 interactions by ∼ 35 kcal mol^-1^. Additionally, while similar in total energies, the ssRNA and ssRNA+ADP substrate states have very different local energies, suggesting different protein-RNA contacts. Specifically, the ssRNA substrate state has stronger interactions with P3 (-206 ± 3 kcal mol^-1^) and P4 (-155 ± 3 kcal mol^-1^) than the other three systems (e.g. ssRNA+ATP, -188.4 ± 0.4 kcal mol^-1^, -139.3 ± 0.6 kcal mol^-1^) and weaker interactions with P1 (ssRNA: -62 ± 2 kcal mol^-1^; ssRNA+ATP: -106 ± 2 kcal mol^-1^). This demonstrates that a bound nucleotide (ATP or ADP) causes a shift in protein-RNA interactions from 3′ (P3 and P4) to 5′ (P1) RNA residues.

#### Hypothesized elementary step in the translocation mechanism

**Fig 7:**
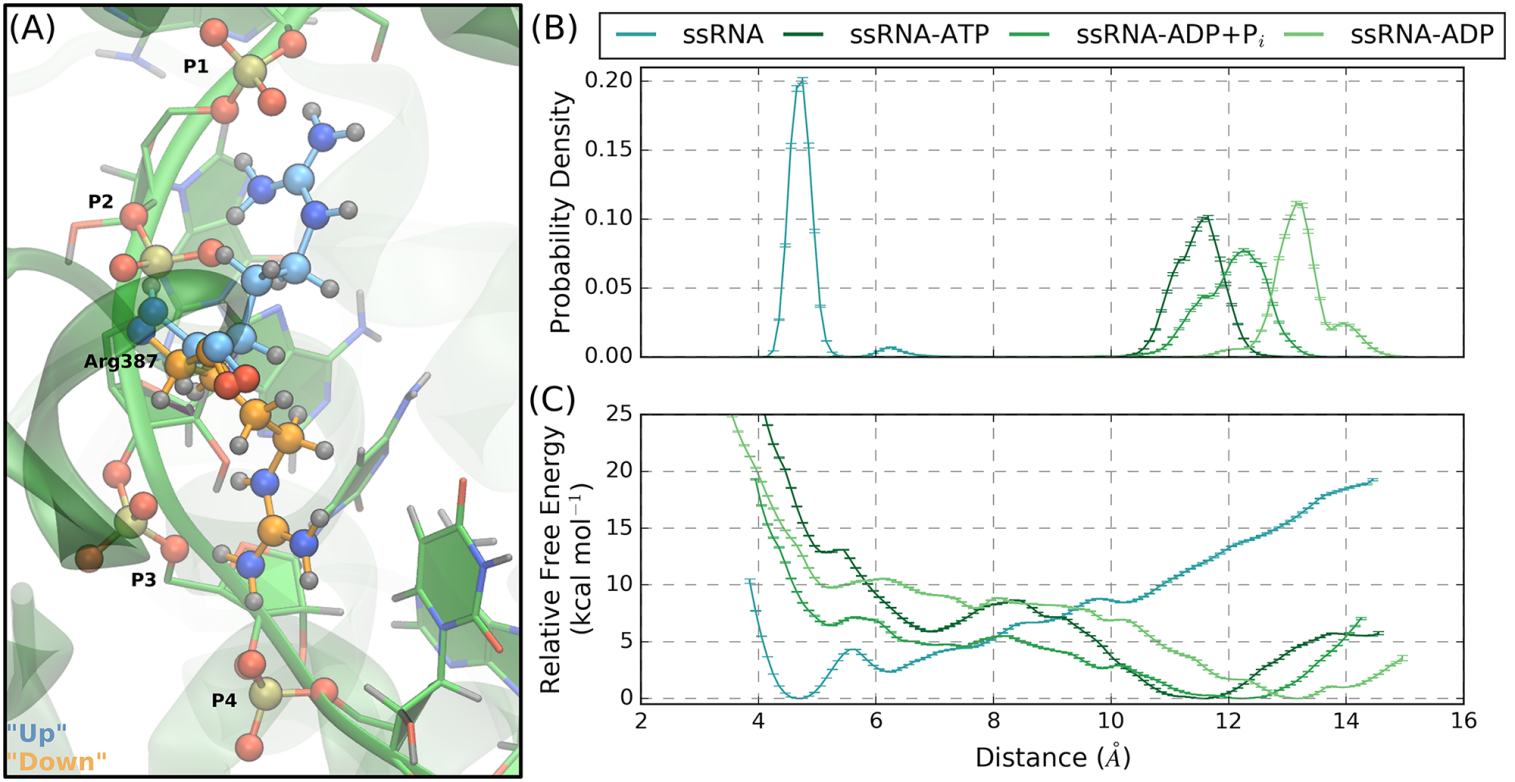
NTPase substrate-dependent interactions between Arg387 of motif IVa and RNA phosphate groups. (A) The guanidinium group of Arg387 is observed to transition from the “up” conformation to the “down” conformation, respectively colored blue and orange. (B) Probability densities and (C) free energy surfaces from the US simulations performed to model the “up” to “down” transition of the Arg387 side chain. Short collective variable distances correspond to the “down” conformation. As emphasized by the line colors, the trend of these results show that ssRNA favors the “down” conformation while the other substrate states favor the “up” conformation, suggesting a NTPase substrate-dependence of the Arg387 conformational states.

Visual analysis of the protein-P3 and -P4 interactions in the ssRNA simulation highlighted a rare event where the guanidinium side chain of Arg387 (motif IVa) transitions from coordinating P1 and P2 to P3 and P4. The two conformations of this event are depicted in Fig 7(A): the “up” conformation (colored blue) has the guanidinium group coordinating P1 and P2 while, in the “down” conformation (colored orange), the side chain coordinates P3 and P4. During the ssRNA+ATP, ssRNA+ADP+P_i_, and ssRNA+ADP simulations, Arg387 is stable in the “up” conformation. In the ssRNA simulation, the “up” to “down” transition occurs once, after which no reverse transitions occur. Furthermore, after Arg387 transitions to the “down” conformation, two concerted events are observed to occur: (1) Lys388 (motif IVa) coordinates P1 and P2, taking up Arg387’s previous position, and (2) P4 partially unbinds from the RNA-binding cleft.

Arg387 and Lys388 are highly conserved residues in the NS3/NPH-II subfamily of SF2 helicases and have been classified as motif IVa residues (positioned at the N-terminus of *α*2′). Lys388 is more solvent exposed than Arg387 and fluctuates around the phosphate groups of the RNA. The *α*2′ secondary structure is generally solvent exposed with its dipole-axis pointing towards P2. Historically, an arginine residue observed to coordinate adjacent phosphate groups of RNA has been termed an arginine fork [92, 93]. Arg387 is one such arginine fork that has been observed to have functional importance to the helicase functions of NS3h. In previous studies of HCV NS3h, the analogous arginine residue (Arg393) was mutated to an alanine, resulting in abrogation of nucleic binding, translocation, and unwinding functions of the mutant HCV NS3h [94]. A recent crystal structure of the Zika virus NS3h in complex with an RNA (PDB ID: 5GJB) has been reported where the analogous motif IVa arginine (Arg388) is positioned in the “down” conformation [95]. This crystal demonstrates that the Arg387 transition observed during DENV NS3h simulation corresponds to a realistic conformation of NS3h:RNA complexes. Furthermore, the high sequence conservation of Arg387 and the mutational results from HCV NS3h suggest that this motif IVa arginine fork binds the RNA and plays an important role in the helicase functions of NS3h.

Due to the concerted nature of the Arg387 transition and the unbinding of P4, we propose that the observed “up” to “down” transition is potentially an elementary step in the translocation mechanism of NS3h, where the guanidinium side chain conformations are NTPase substrate-dependent. Therefore, we investigate the thermodynamics of the side chain conformational states using one-dimensional US simulations for the ssRNA, ssRNA+ATP, ssRNA+ADP+P_i_, and ssRNA+ADP systems. The biased collective variable is the distance between the central carbon of the Arg387 guanidinium group to the phosphorous atom of P4, where the “up” and “down” conformations correspond to long and short distances, respectively. The resulting probability densities and relative free energy surfaces from the US simulations are shown in Fig 7(B) and 7(C), respectively. The relative changes in free energy between the “up” and “down” side chain conformations (Δ G_US_) are -7.52±0.05 kcal mol^-1^, 13.885±0.05 kcal mol^-1^, 7.45±0.06 kcal mol^-1^, and 10.91±0.05 kcal mol^-1^ for the ssRNA, ssRNA+ATP, ssRNA+ADP+P_i_, and ssRNA+ADP systems, respectively. Therefore, the Arg387 side chain states are observed to have a NTPase substrate-dependence where the ssRNA system energetically favors the “down” conformation while the ssRNA+ATP, ssRNA+ADP+P_i_, and ssRNA+ADP substrate states favor the “up” conformation. Unbinding of P4 is not observed for US windows corresponding to the “down” conformation in the ssRNA+ATP, ssRNA+ADP+P_i_, and ssRNA+ADP US simulations.

These results support the hypothesis that the Arg387 side chain conformational states are NTPase substrate-dependent and exemplify a large shift in protein-RNA interactions. Considering the full NTPase cycle (Fig 1), Arg387 thermodynamically favors the “down” conformation in the ssRNA substrate state where the guanidinium group coordinates P3 and P4. Subsequently moving through the NTPase substrate states, Arg387 is expected to transition to the “up” conformation and coordinate P1 and P2. Therefore, transitions between the Arg387 conformational states are predicted to impart a 3′ to 5′ direction to interactions between NS3h and RNA. Furthermore, the concerted events that were observed to followed the Arg387 conformational change (Lys388 coordinating P1 and P2; partial unbinding of P4) are novel events that have potentially important implications for the translocation of NS3h along the phosphodiester backbone of ssRNA. These results support the hypothesis that the Arg387 conformational states represent states of a NTPase substrate-dependent, elementary step in the unidirectional translocation mechanism of NS3h.

### Allosteric pathways

The current view of allosteric regulation focuses on signal transduction through complex, 3-dimensional networks, brought about by intrinsic structural and/or dynamic changes along pathways connecting two distal, non-overlapping active sites [96–98]. These allosteric pathways are described by coupled short-range, residue-residue interactions that lead to long-range correlations. In the previous two sections, RNA-induced and NTPase substrate-induced structural rearrangements have been presented. In this section, these allosteric structural changes are absorbed into a unified description of the allosteric pathways connecting the RNA-binding cleft with the NTPase active site.

Dynamic network analyses, such as residue-residue correlations, have been used to identify allosteric pathways within proteins from simulation [96–100]. A growing body of literature has highlighted the functional importance of such pathways as well as the fundamental residue-residue interactions leading to their emergence [96–102]. We report here residue-residue distance correlation analyses that are used to identify the allosteric pathways present within the DENV NS3h protein. Focus is given to the motifs discussed in the previous sections (*α*2, motifs II and IVa) due to the observed structural rearrangements. Additionally, the correlation heat maps are used to identify segments of the protein that experience strong correlations with numerous other regions of the protein, such as motif V. While motif V does not experience substrate-induced structural rearrangements, the strong correlations between motif V and motifs in both the NTPase active site and RNA binding cleft are hypothesized to have functional importance in the signal transduction mechanism of allosteric regulation. Unlike the previous two sections, comparisons between substrate states (RNA-bound and NTPase substrate-bound) are not considered here. Instead, focus is given to the discussion of the residue-residue distance correlation analysis of the ssRNA+ATP substrate state.

#### Correlations between motifs

**Fig 8:**
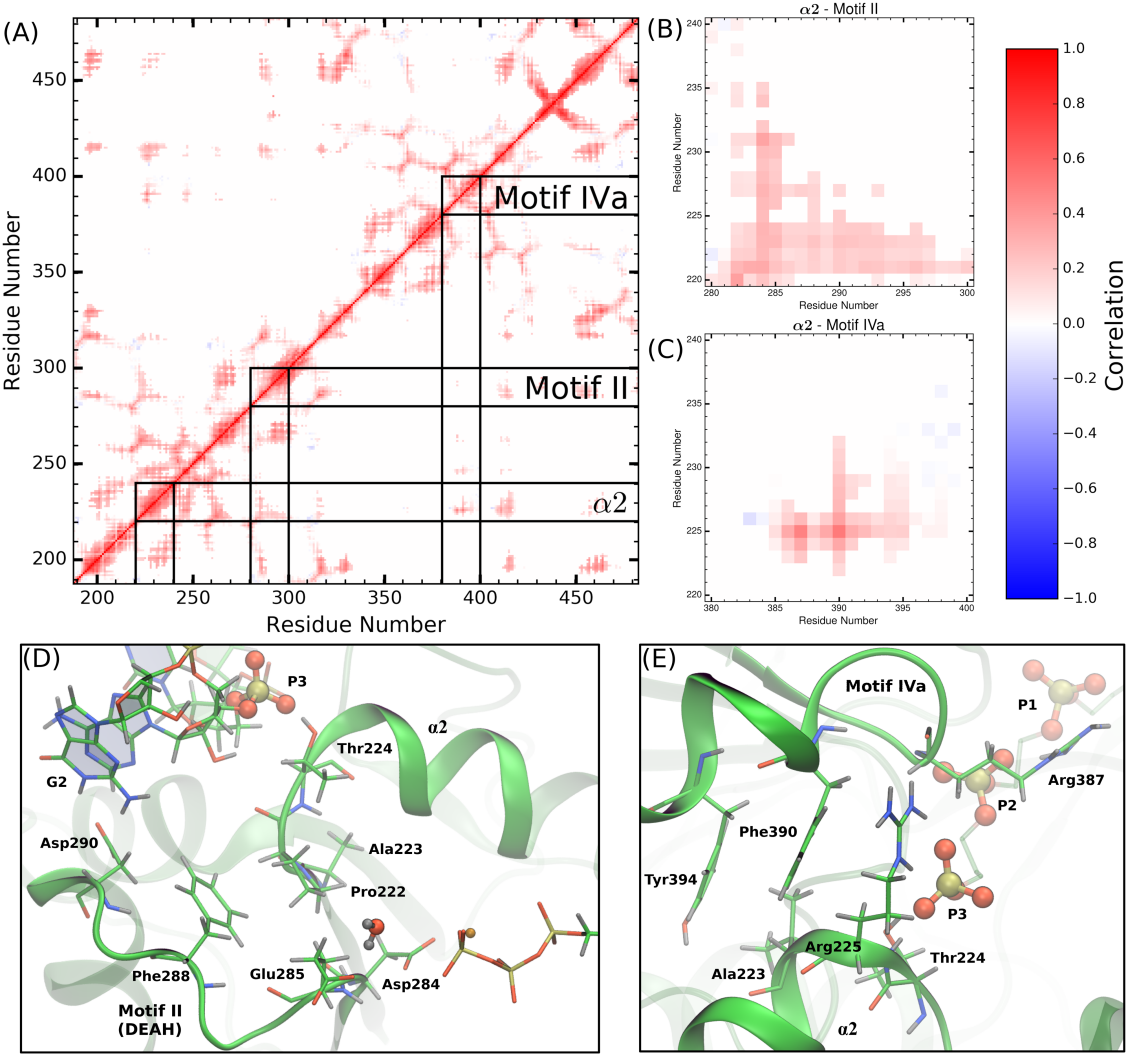
Correlated motions of protein motifs observed to experience RNA- or NTPase substrate-induced allostery. (A) The average COM-COM residue pair correlation heat map for the ssRNA+ATP system abridged with a distance cutoff of 15 Å. Lines drawn highlight the structural motifs discussed in the previous two sections (*α*2, motifs II and IVa). Panels (B) and (C) are magnifications of off-diagonal regions in (A) that correspond to the correlations between *α*2 and motif II or motif IVa, respectively. Hotspots within these regions identify the short-range residue-residue interactions that couple the structures. Panels (D) and (E) provide structural depiction of these residue-residue interactions.

Fig 8(A) shows the average residue-residue distance correlation heat map for the ssRNA+ATP substrate state, where residue pairs with strong correlated and anti-correlated motions are colored red or blue, respectively. The correlation heat map is abridged by setting correlation values to zero if the average COM-COM distance of residue pairs is greater than 15 Å. This simplification limits the correlation analysis to residue pairs within a close proximity, thereby identifying the short-range interactions that build up to the pathways connecting the RNA-binding cleft and the NTPase active site. Correlation heat maps for the other substrate states are presented in SI (Figs I-M in S1 Text).

As expected, there is strong correlation along the linear sequence of residues, as seen in Fig 8(A) along the diagonal. Secondary structures experience more extended linear sequence correlations than non-structured regions (thickness of diagonal sections). The RecA-like *β*-sheets in subdomains 1 (residues 188 to 326) and 2 (residues 327 to 481) produce the honeycomb patterns observed in the heat map, due to the extremely stable tertiary structure observed in NS3h. Lines drawn on Fig 8(A) highlight a range of 20 residues centered on *α*2, motifs II and IVa.

Panels (B) and (D) of Fig 8 highlight the *α*2/motif II off-diagonal region of the heat map and the structural features leading to the observed correlations between these two segments. Specifically, Asp284 and Glu285 of motif II experience correlated motions with most of *α*2 (residues 220 to 231) and especially strong correlations with Ala222, Pro223, and Thr224. Thr224 directly coordinates phosphate 3 of the RNA substrate. Therefore, panels (B) and (D) highlight a pathway through Glu285, Ala222, Pro223, and Thr224 that connects residues of paramount importance in the hydrolysis reaction (Glu285) with residues that directly coordinate the phosphodiester backbone of the RNA (Thr224).

In a similar fashion, the motif IVa structure is observed to have strong correlations with residues in *α*2, as shown in panels (C) and (E) of Fig 8. Phe390 and, to a lesser extent, Tyr394 of motif IVa are observed to have strong correlated motions with residues 222 to 232 of *α*2. Alternatively, from the perspective of *α*2, the guanidinium group of Arg225 has strong and maintained electrostatic interaction with the backbone carbonyl group of Arg387. Through this short-range interaction, Thr224, Arg225, and Ala226 experience correlated motions with residues 385 to 395 of motif IVa. As observed in Fig 8(E), the side chains of Arg225, Phe390, and Tyr394 are positioned in a *π* stacking-like structure, bookended by RNA phosphate 3 as well as residues in the NTPase active site (residues not shown for clarity but can be viewed in trajectories provided at https://hdl.handle.net/10217/186709).

Direct coupling between motifs II and IVa is minimal due to the large distances between the residues in both motifs, as shown in the respective off-diagonal region of Fig 8(A). Rather, short-range interactions between residues in *α*2 (Ala222, Pro223, Thr224, Arg225) with residues of motifs II (Asp284, Glu285) and IVa (Arg387, Phe390, Tyr394) act as channels through which free energy released from the NTPase cycle or helicase functions can be transferred from the NTPase active site to the phosphodiester backbone of the RNA (and vice versa). Structural or dynamic perturbation of these pathways, via mutation or small molecule binding, are hypothesized to affect the efficiency of energy transduction.

#### Motif V

**Fig 9:**
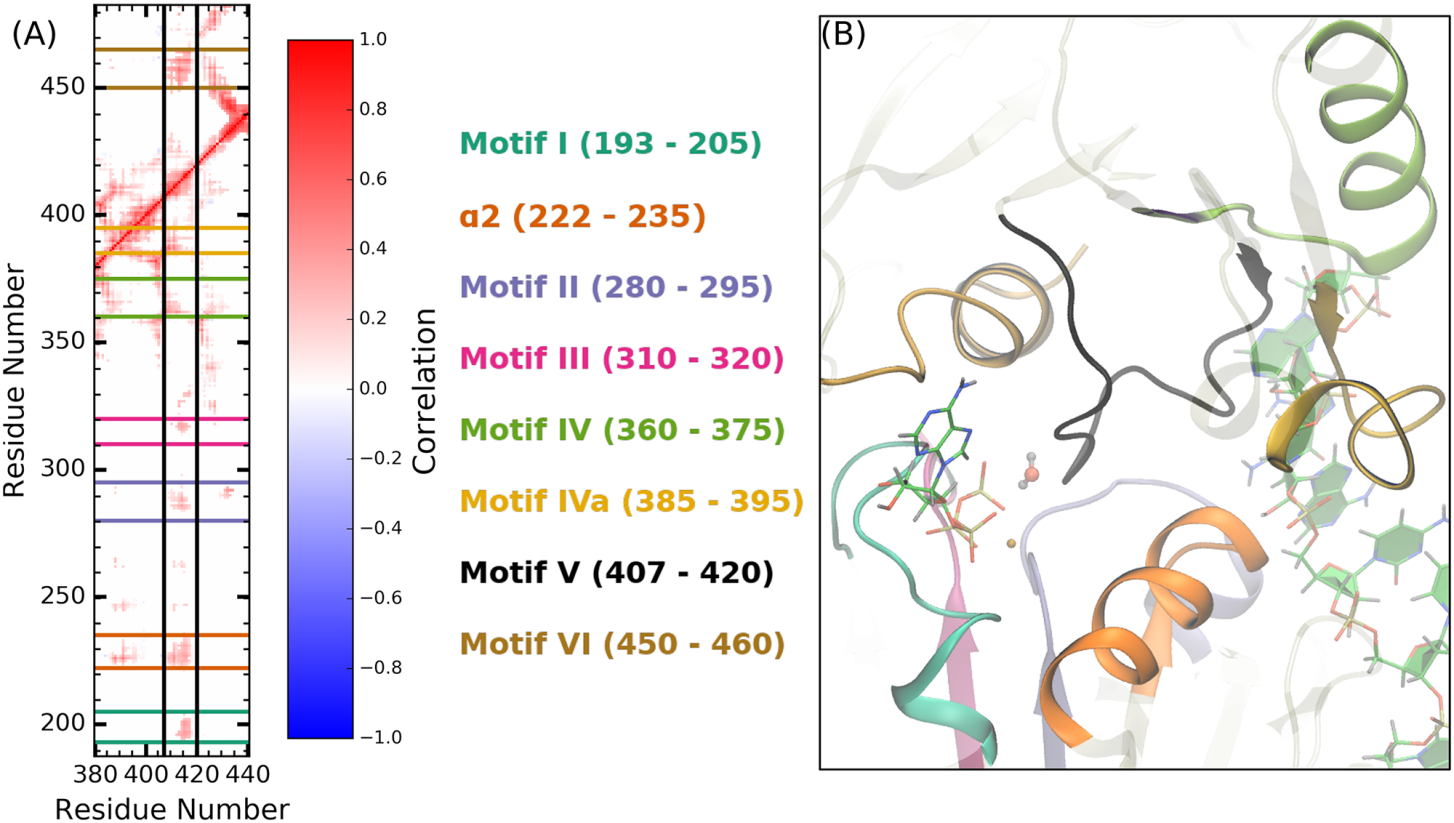
Motif V is a highly correlated and centralized structure within subdomains 1 and 2. (A) Vertical segment of the ssRNA+ATP correlation heat map focusing on motif V (residues 407 to 420). Conserved motifs that have strong correlations with motif V are highlighted by horizontal lines on the heat map, colored as shown in the legend. (B) ssRNA+ATP exemplar structure depicting the central position of motif V in relation to the NTPase active site and the conserved motifs highlighted in panel (A). The ATP and lytic water molecules are shown to highlight the proximal location of motif V residues with respect to the NTPase active site.

The residue-residue correlation analyses are used to identify structural regions of NS3h that exhibited strong correlations with other regions of the protein. Particularly, this analysis highlighted residues 407 to 420 of motif V. Fig 9(A) shows the motif V correlation heat map section for the ssRNA+ATP system, where lines are drawn to highlight the strong coupling between these residues and residues of motifs I, II, III, IV, IVa, VI as well as *α*2. Structurally, the highly correlated nature of motif V is explained by the position of these residues in relation to the NTPase active site, RNA-binding cleft, and protein residues important in either active site, as shown in Fig 9(B). Motif V consists of residues in subdomain 2 and has a complex secondary structure (loop into short *α*-helix into loop). The backbone amide of Gly414 actively coordinates the lytic water of the hydrolysis reaction. The alcohol group of Thr408 coordinates phosphate 2 of the RNA. Therefore, the linear sequence of motif V is a direct pathway connecting the NTPase active site with the RNA-binding cleft. Limited structural changes are observed for this motif in the presence or absence of RNA or NTPase substrates. Rather, motif V is observed to have strongly correlated motions with the previously mentioned structural regions for all substrate states. Similar to the previously discussed pathways, motif V is hypothesized to be another pathway for free energy transduction from one active site to the other.

### Conclusion

Through analyses of the reported simulations, molecular observables of RNA- and NTPase substrate-induced allostery were identified. Specifically, an RNA bound within the RNA-binding cleft affects the dynamics and positioning of water molecules within the NTPase active site. This allosteric influence is conferred from the RNA-binding cleft to the hydrolysis active site through structural rearrangements of L*β*3*β*4, *α*2, and motif II. These RNA-induced structural changes lead to an entropic destabilization of the NTPase active site as well as a direct destabilization of the lytic water. Inspired from these results, electronic structure calculations were used to investigate the energetics of NTP hydrolysis reaction. The energetic landscapes obtained from the DFT calculations demonstrate that RNA decreases the activation barrier as well as affects the mechanism of the hydrolysis reaction. Combining these results into a kinetic model allowed for the calculation of a theoretical RNA-stimulated NTPase activity enhancement factor of 150, which qualitatively matches the experimentally observed enhancement factor. Therefore, results from MD and DFT calculations provide novel, multiscale insight into the RNA-induced allosteric effects that stimulate the catalysis of the NTP hydrolysis reaction in DENV NS3h.

Unlike RNA, the NTPase substrates are smaller perturbations to the NS3h structure and dynamics. Protein-RNA interaction energies were used to investigate the NTPase substrate-dependence of protein-RNA contacts in the unbiased MD simulations. From these analyses, the protein-RNA phosphodiester backbone interactions were observed to be NTPase substrate-dependent. The presence of the *γ*-phosphate (or P_*i*_) of the NTPase substrate was observed to strengthen the protein-RNA contacts. Furthermore, the localized nonbonding interaction energies demonstrate a large shift in protein-RNA contacts, originating in part from the side chain conformational states of Arg387. Results from US simulations demonstrate that the Arg387 side chain conformational states exemplify NTPase substrate-dependent protein-RNA interactions. With the purview of the NTPase cycle, transitions between conformational states leads to 3′ to 5′ translocation. Therefore, we hypothesize that the transition between Arg387 side chain conformations is an elementary step in the unidirectional translocation mechanism of NS3h along the phosphodiester backbone of RNA.

Finally, consideration of these allosteric effects independent of one another provides an incomplete picture of the biophysics of the NS3h protein. Residue-residue correlation analyses were used to identify structural regions of the protein that experienced correlated motions with other regions. These analyses were used to describe the allosteric pathways that connect *α*2 with motifs II and IVa. The short-range, residue-residue interactions were presented that connect the RNA-binding cleft to the NTPase active site. Furthermore, the correlation heat maps allow for identification of regions of the protein that experience strong correlated motions with numerous other regions. Motif V is one such example, where the segment of 13 residues has strong coupled motions with seven other motifs in subdomains 1 and 2. This highly correlated nature suggests that motif V functions as a centralized communication hub that connects distal portions of the protein structure.

Complete modeling of a revolution of the NTPase cycle in the DENV NS3h presents a significant challenge for current computational methodologies. Rather, we have divided the cycle into equilibrium substrate states and dynamic events where the protein transitions from one substrate state to another. The simulations reported have modeled the important NTPase cycle substrate states, leading to novel insights into the function and underlying biophysics of the DENV NS3h enzyme with focus given to the allosteric connections between the RNA-binding cleft and NTPase active site. We hypothesize that the observed allosteric effects and pathways have important roles in the transduction of energy from one active site to the other during the dynamic events of the NTPase cycle. Therefore, the results reported have laid an initial foundation for theoretical investigations into the dynamic events of the NTPase cycle.

Beyond further theoretical modeling of NS3h, transverse relaxation-optimized spectroscopy (TROSY) NMR, mutational biochemical studies, and targeted small molecule binding experiments can be envisioned to test the hypothesis and results presented. Nuclear magnetic resonance has been previously used to study dynamics within isolated HCV NS3 helicase subdomains [103–105], but the size of the full dengue NS3h domain is too large for traditional NMR approaches due to line width increasing with increased molecular mass. However, TROSY NMR has been developed that may allow for experimental monitoring of fast NS3h dynamics [106]. We anticipate that perturbation of the wild-type structure or dynamics of the allosteric pathways in NS3h will lead to abrogation of NTPase and/or helicase functions, and are currently developing assays to test this hypothesis with dengue NS3h. Residues active in the allosteric pathways are viable targets for mutational studies where varying a specific amino acid residue is expected to alter the short-range, residue-residue interactions and lead to a destabilization of the pathway connecting the two active sites. This is hypothesized to result in reductions in enzymatic activity and would be observable in our biochemical assays. Additionally, these pathways are viable targets for theoretical and experimental small molecule drug docking experiments with focus given to molecules that disrupt the residue-residue interactions along the pathway. Co-crystallization of specific conformation-binding molecules may lock NS3h into transition-state conformations that can help verify our computational studies. Molecular candidates also have the potential for inhibiting NS3h function during replication and being specific to the NS3/NPH-II subfamily of SF2 helicases.

## Supporting information

S1 File Supplementary information file. The file includes further details about simulation protocol and analyses performed on the MD simulations as well as structural figures of the atoms included in the electronic structure calculations. Additionally, residue-residue correlation heat maps of the Apo, ATP, ssRNA, ssRNA+ADP+P_i_, and ssRNA+ADP substrate states are presented. Cartesian coordinates of the optimized structures of the NTPase active site are tabulated.
(PDF)

S2 File MD simulation parameters and configuration files. Unzipping this file will create a directory containing the parameters and starting structures used for the reported unbiased simulations. Configuration files to run simulation in the AMBER software packages are also provided. Additionally, the structure prep file for inorganic phosphate, P_*i*_, as well as the frcmod.phosaa10 files are included. It is necessary to load these files into leap to obtain the P_*i*_ parameters and structure used in the reported simulations.
(ZIP)
