# Supplementary material for: Allostery in the dengue virus NS3 helicase: Insights into the NTPase cycle from molecular simulations
Source: PLoS Comput Biol. 2018 Apr 16;14(4):e1006103. doi: 10.1371/journal.pcbi.1006103 (PMC5919694; doi:10.1371/journal.pcbi.1006103)
Supplement: S1 File — The file includes further details about simulation protocol and analyses performed on the MD simulations as well as structural figures of the atoms included in the electronic structure calculations. Additionally, residue-residue correlation heat maps of the Apo, ATP, ssRNA, ssRNA+ADP+Pi, and ssRNA+ADP substrate states are presented. Cartesian coordinates of the optimized structures of the NTPase active site are tabulated. (PDF) [file pcbi.1006103.s001.pdf]

Supplementary Information for: Allostery in the dengue virus NS3 helicase: Insights into the NTP hydrolysis cycle from molecular simulations

Russell B. Davidson, Josie Hendrix, Brian J. Geiss, Martin McCullagh

March 30, 2018

1 System Preparation

Molecular Dynamics Simulations Energy minimization of MD starting structures was performed in two steps: 1) 10,000 steps of minimization with the protein, substrates, and crystallographic waters restrained and 2) another 10,000 steps with all atoms unconstrained. Initial heating of each simulation was performed where minimized structures were heated from 0 K to 300 K over 0.5 ns. The heating simulation was performed with 75 kcal mol^{-1} harmonic restraints on all non-solvent atoms. Equilibration of the protein structure at 300 K was performed by slowly releasing the applied restraints over the course of 2 ns. This minimization and heating protocol was used for all reported simulations.

QM structures As mentioned in the article, reactant starting structures for the density functional theory (DFT) calculations were grabbed from the body of MD frames that posses a water molecule in a lytic position. To demonstrate that the initial structures used in the DFT calculations adequately represent the population of frames with lytic waters, the RMSD of protein side chain heavy atoms included in the QM region was calculated for all MD frames, referenced against the DFT starting structures. For the ATP substrate state, the average RMSD for this selection is 1.2 $\pm$ 0.4 Å. For the ssRNA+ATP substrate state, the average RMSD for this selection is 1.2 $\pm$ 0.3 Å. The high relative standard deviation of these values is associated with fluctuations of Ala316 atoms during the MD simulations.

The QM region consists of 138 atoms corresponding to the methyl triphosphate, Mg^{2+} cation, six water molecules, and ten amino acid residues closest to the triphosphate tail of the ATP molecule (shown in Figure A). The amino acid residues were truncated at various positions with hydrogen atoms. For example, a hydrogen atom was added to the backbone amide of Pro195 as well as the C_α atom of Gly196. Lys199 was truncated at C_ϵ atom. For Thr200, the alcohol group is truncated with a hydrogen, thereby bringing the number of water atoms to seven and number of amino acid residues down to nine (as reported in the article). Glu285 is truncated at the C_β atom. Ala316 is truncated on the backbone amide group and C_α atoms. Similarly, Gly414 is truncated at the backbone amide and carbonyl atoms. Gln456 is truncated at the C_γ atom. Both Arg460 and Arg463 are truncated at the C_γ

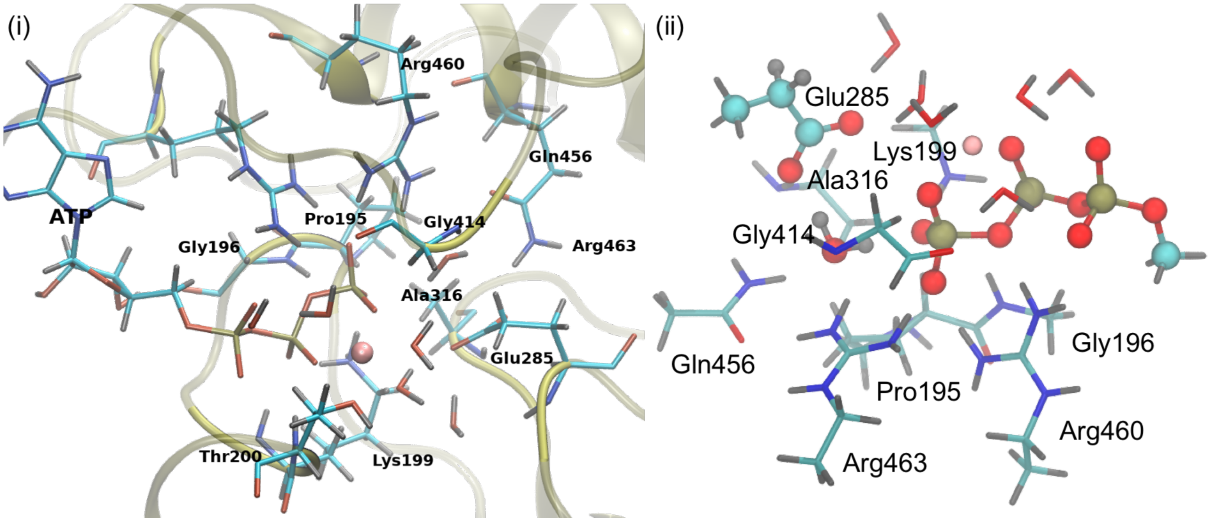

Figure A: Structural representation of the QM region. (i) The QM region within the broader protein structure. Residue labels included in the QM region are provided. Thr200 is included because the alcohol group of the side chain was replaced with a water molecule in the electronic structure calculations. (ii) Depiction of the QM region with full truncation used in the electronic structure calculations. Atoms highlighted with the CPK representation are shown in the energy landscape (Fig 5) in the article.

atoms. These truncations maintain the coordination sphere of Mg^{2+} as well as the important contacts between the triphosphate tail and amino acid functional groups. Waters included in the QM calculations were chosen due to their proximity to the terminal phosphoanhydride bond as well as the Mg^{2+} cation.

2 Data Analysis

Equilibration and Convergence of Simulations Global and local protein structural metrics (e.g. radius of gyration, RMSD referenced against the respective crystal structures; Figures B to D) are used to determine that the protein structure in each simulation had adequately converged away from the crystal structure after 200 ns of simulation. Further convergence of the simulations is measured by separating the full trajectories into 50 ns windows that are treated as independent trajectories for the purpose of convergence and error analyses. Comparisons between these windows are made to identify long-timescale changes occurring during the microsecond-long trajectories. Additionally, for analysis metrics discussed in the paper, averages of these windows are used to determine the standard error of the mean for the full dataset. For the substrate states with a pair of microsecond-long trajectories (ATP and ssRNA+ATP), each replica is given 200 ns to equilibrate from the starting structures, after which analysis results from both replicas are combined into the reported ensemble averages. Error analysis of these averages include the 50 ns windows from both replicas.

Alignment Landmarks The β-sheets in the RecA-like subdomains of DENV NS3h (subdomains 1 and 2) are observed to be extremely stable during the reported MD simulations

as well as across all substrate states. Therefore, this collection of secondary structures acts as a good reference point for analyzing structural and dynamic changes in the rest of the protein. Specifically, the C_α atoms of the residues making up these secondary structures (listed in Table A) are used as the landmark for all analyses requiring structural alignment of the protein. Figure E shows the aligned RMSD of this landmark for all trajectories as referenced against the ssRNA+ATP crystal structure (PDB ID: 2JLV). Similarly, Figure F shows the RMSD of all heavy-atoms for residues comprising these β-sheets, referenced against the ssRNA+ATP crystal structure and using the alignment landmark. Structurally, these β-sheets are structurally conserved across the SF1 and SF2 helicase families and so we hypothesize that this landmark can be efficiently used for broad structural comparisons within these families.

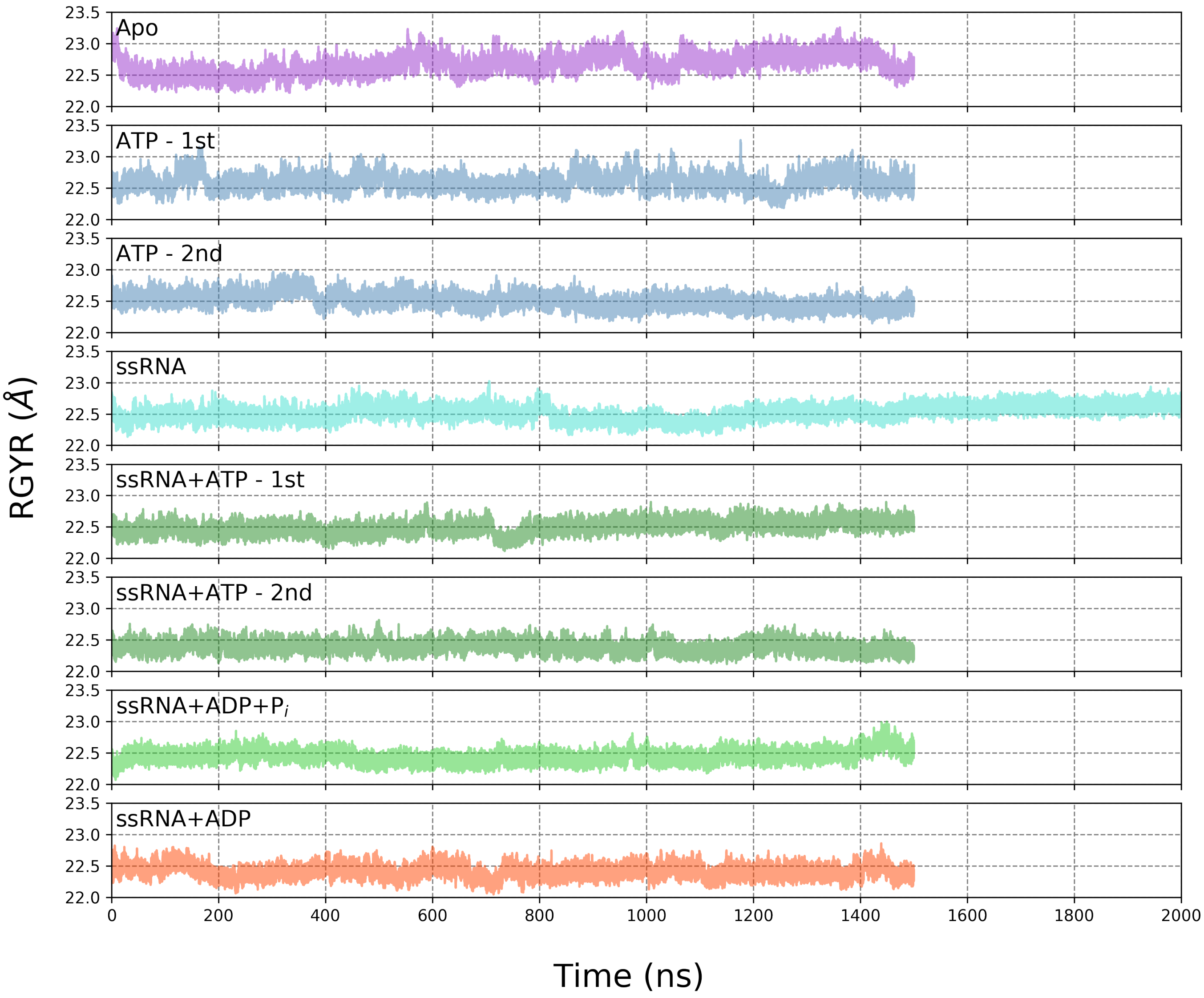

Figure B: RGYR of the protein and substrates. As seen through this metric (e.g. Apo, ssRNA+ADP+P$_i$, and ssRNA+ADP), the RGYR metric deviates from the starting values during the first 200 ns of simulation.

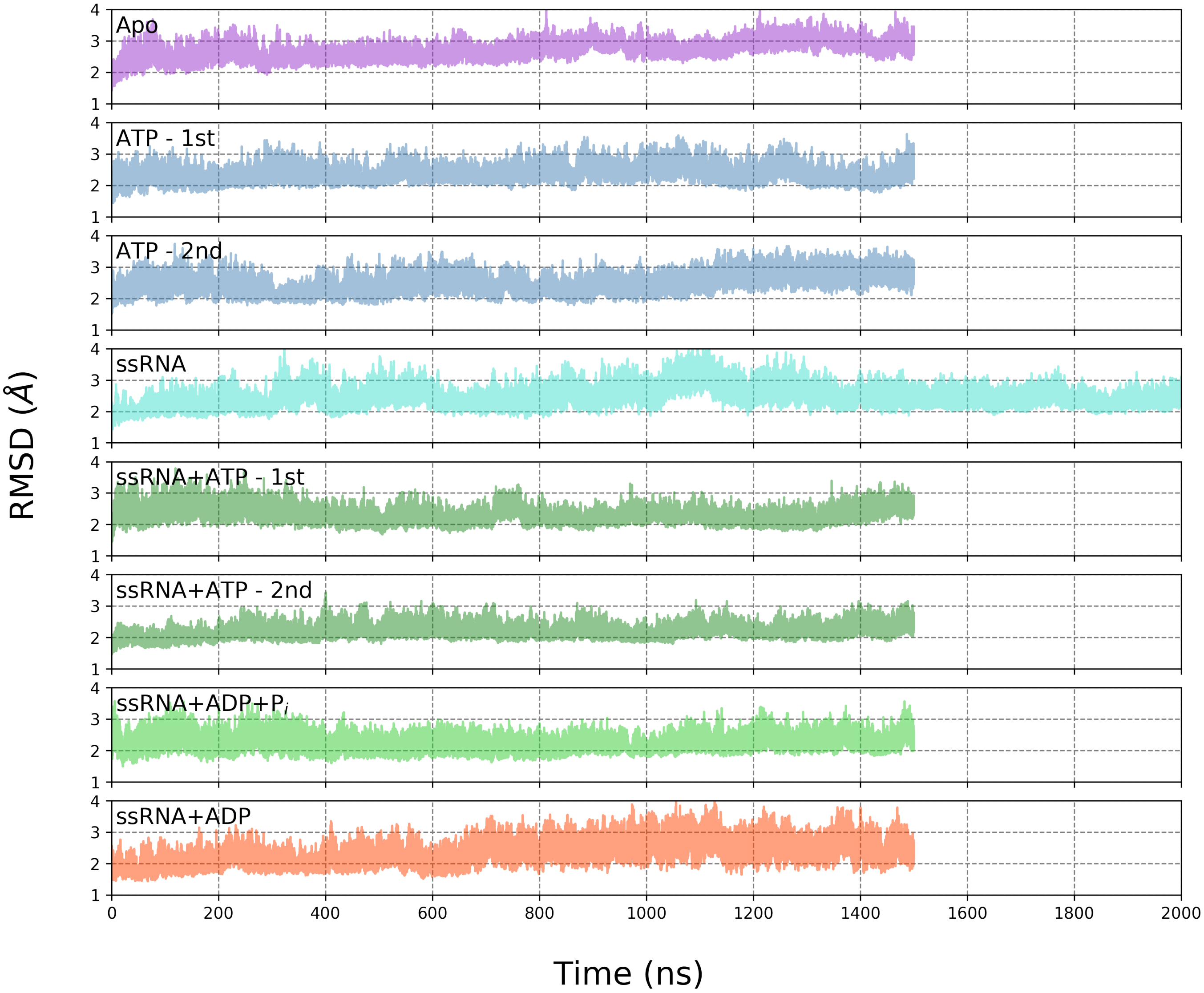

Figure C: RMSD of all heavy atoms of the protein, referenced against the respective crystal structures. Before the calculation, every frame is aligned to the β-sheets discussed in the Alignment Landmarks section.

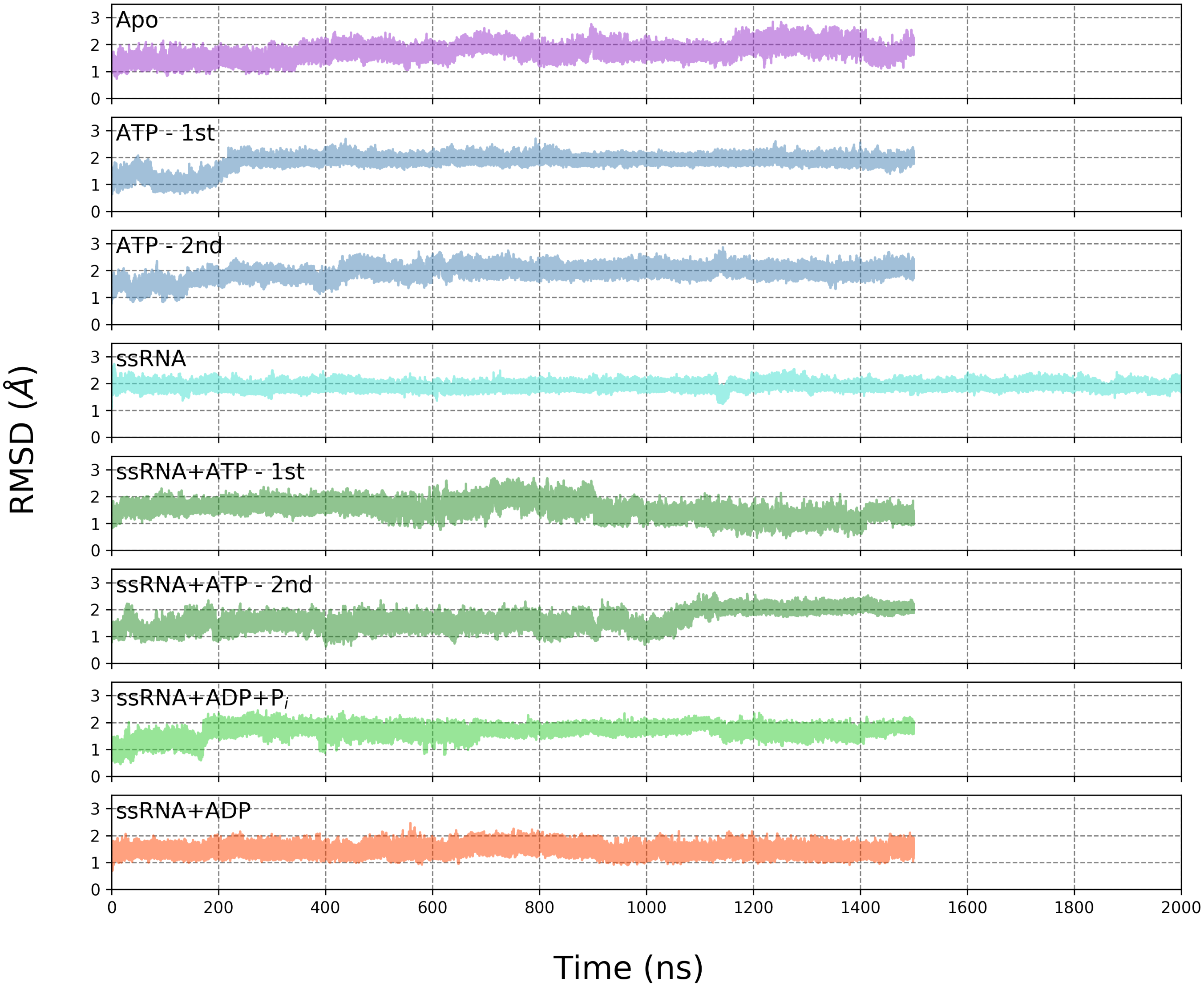

Figure D: RMSD of all heavy atoms of motif II residues (residue IDs: 284 to 291), referenced against the respective crystal structures. Before the calculation, every frame is aligned to the β-sheets discussed in the Alignment Landmarks section.

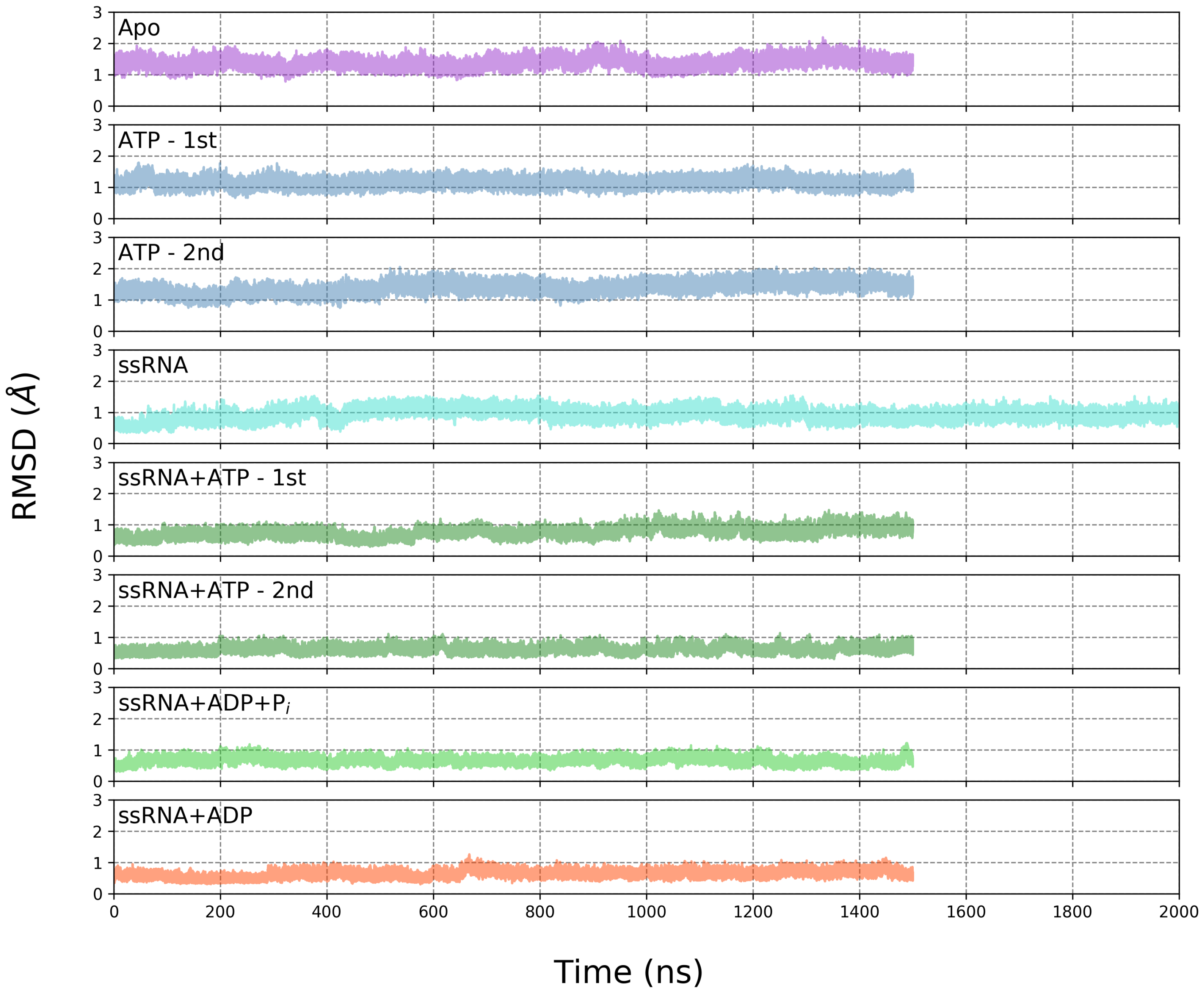

Figure E: RMSD of the Cα atoms of the RecA-like β-sheets referenced against the ssRNA+ATP crystal structure (PDB ID: 2JLV) with the alignment landmark applied. Residue numbers for this atom selection are provided in Table A. Generally, RMSD values are small and uniform for all simulations and thus support the use of this alignment landmark.

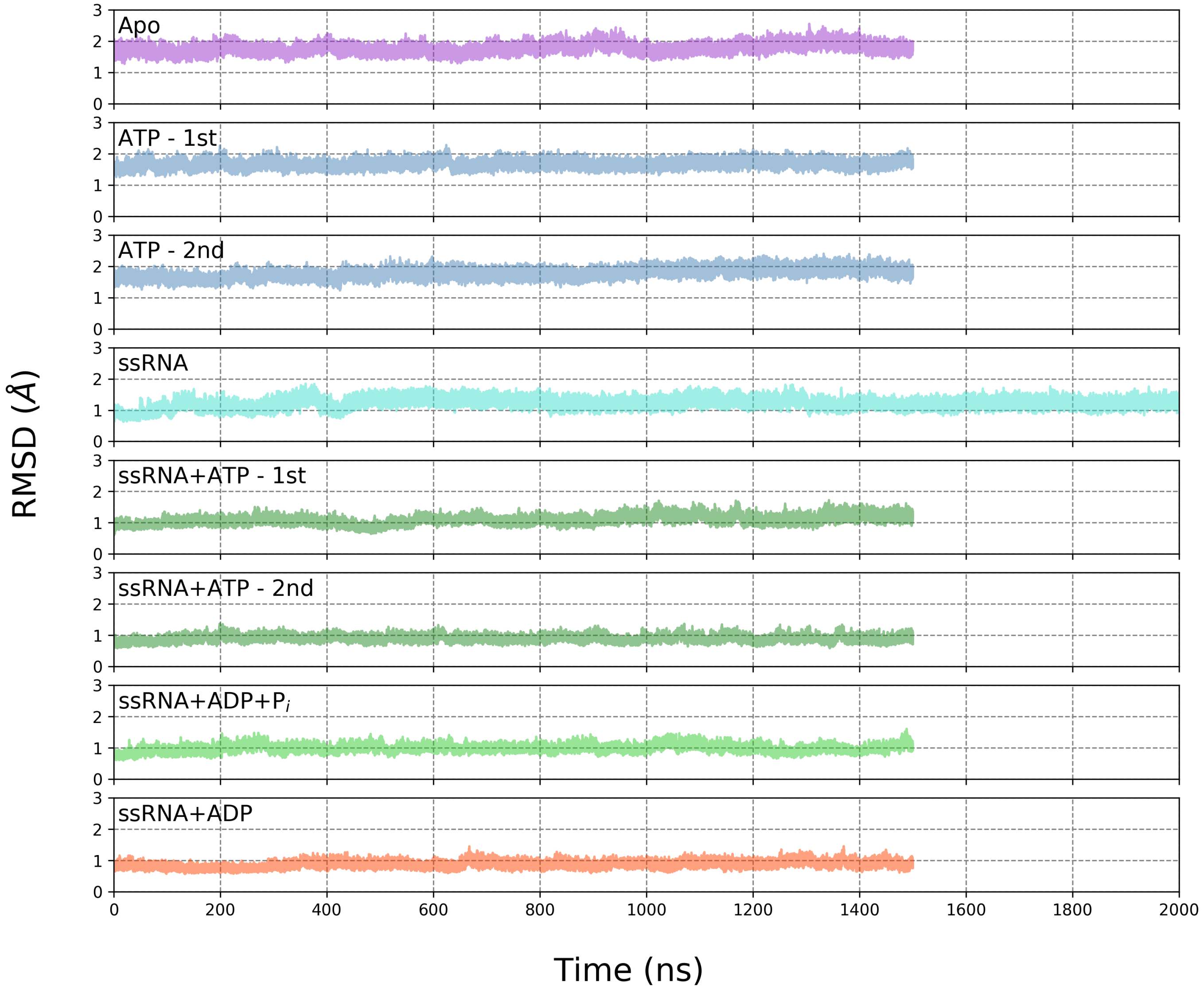

Figure F: RMSD of the RecA-like β-sheets referenced against the ssRNA+ATP crystal structure (PDB ID: 2JLV) with the alignment landmark applied. Residue numbers for this atom selection are provided in Table A.

Table A: Residue numbers for each β-sheet in the alignment landmark.

Secondary Structure	Residue Numbers
$\beta\mathbf{1}$	187-192
$\beta\mathbf{2}$	217-222
$\beta\mathbf{3}$	240-242
$\beta\mathbf{4}$	257-261
$\beta\mathbf{5}$	279-283
$\beta\mathbf{6}$	309-314
$\beta\mathbf{1'}$	332-336
$\beta\mathbf{2'}$	357-361
$\beta\mathbf{3'}$	381-385
$\beta\mathbf{4'}$	403-407
$\beta\mathbf{5'}$	420-425
$\beta\mathbf{6'}$	470-474

Exemplar Structures All structural figures presented in this manuscript were created in a consistent fashion: for each substrate state, a single frame from the total number of frames was chosen to be the "exemplar" structure for the substrate state. The exemplar structure corresponds to the frame with the minimum RMSD value of all protein, heavy atoms referenced against the average structure of the equilibrated portion of the microsecond-long trajectories.

Definition of the Center of the NTPase Active Site The center of the NTPase active site was determined by measuring the center of mass of a subset of protein residues that are prominent within the active site. These residues (numbers: 194 to 202, 227, 230, 231, 284, 285, 314, 316, 326, 412 to 416, 455, 456, 459, 460, and 463) were identified using protein-ATP(ADP) distance analyses and visual analysis of trajectories. Waters within an 8 Å radius of the COM coordinate are considered to be within the active site and are analyzed further.

Definition of Lytic Waters Of the waters within the NTPase active site, a lytic water was defined as a water with an P_γ-O_{wat} distance less than 5 Å and $O_{\beta,\gamma}$-P_γ-O_{wat} angle greater than 155°. The definition of the angle cutoff is supported by the electronic structure results, where the lytic water in the ssRNA+ATP and ATP substrate state reactant structures have an $O_{\beta,\gamma}$-P_γ-O_{wat} angle of 161° and 157°, respectively. These geometric cutoffs are used to identify frames in the ATP and ssRNA+ATP MD simulations that have water(s) near ideal lytic positions. The reported probabilities in section 3.1.3 were calculated by counting the number of frames with a lytic water and dividing by the total number of frames. The error for this measurement was approximated by assuming a Poisson distribution for counting experiments.

The two-dimensional heat maps of NTPase active site water positions projected on the P_γ-O_{wat} distance and $O_{\beta,\gamma}$-P_γ-O_{wat} angle are presented in Figures G and H.

Water Mean Squared Displacement and O-H Bond Autocorrelation The mean squared displacement (MSD) and O-H bond autocorrelation analyses were measured for waters within the NTPase active site. To obtain the necessary time resolution, 1 ns trajectories were initiated from restart files every 50 ns. Frames were written every 0.2 ps, providing 5000 frames per trajectory to calculate the MSD and O-H bond autocorrelation metrics as a function of time. Rather than use the β-sheet alignment landmark, the NTPase active site residues were used as the alignment landmark for this analysis thus providing a more localized alignment. Both metrics were averaged over the post-equilibration trajectories (26 trajectories for each system except for the ssRNA system which had 36 trajectories).

Nonbonding Interaction Energies The linear interaction energy (*lie*) function of AMBER14 cpptraj was used to calculate the pairwise sum of Lennard-Jones (LJ) and short-range, unshielded electrostatic energies between the protein and the RNA substrate.[1] A 12 Å distance cutoff was applied for both LJ and electrostatic energies. Reported errors in the interaction energies were calculated by measuring the standard error of the averages of the 50 ns windows (discussed above).

Correlation Analyses The calculation and utility of residue-residue distance correlation analyses have been thoroughly discussed in the literature.[2, 3, 4, 5] Nodes in the correlation matrix were defined as the center of mass of each residue. The correlation heat map is abridged by applying a distance cutoff of 15 Å where correlation values are set to zero if the average COM-COM distance is greater than the cutoff.

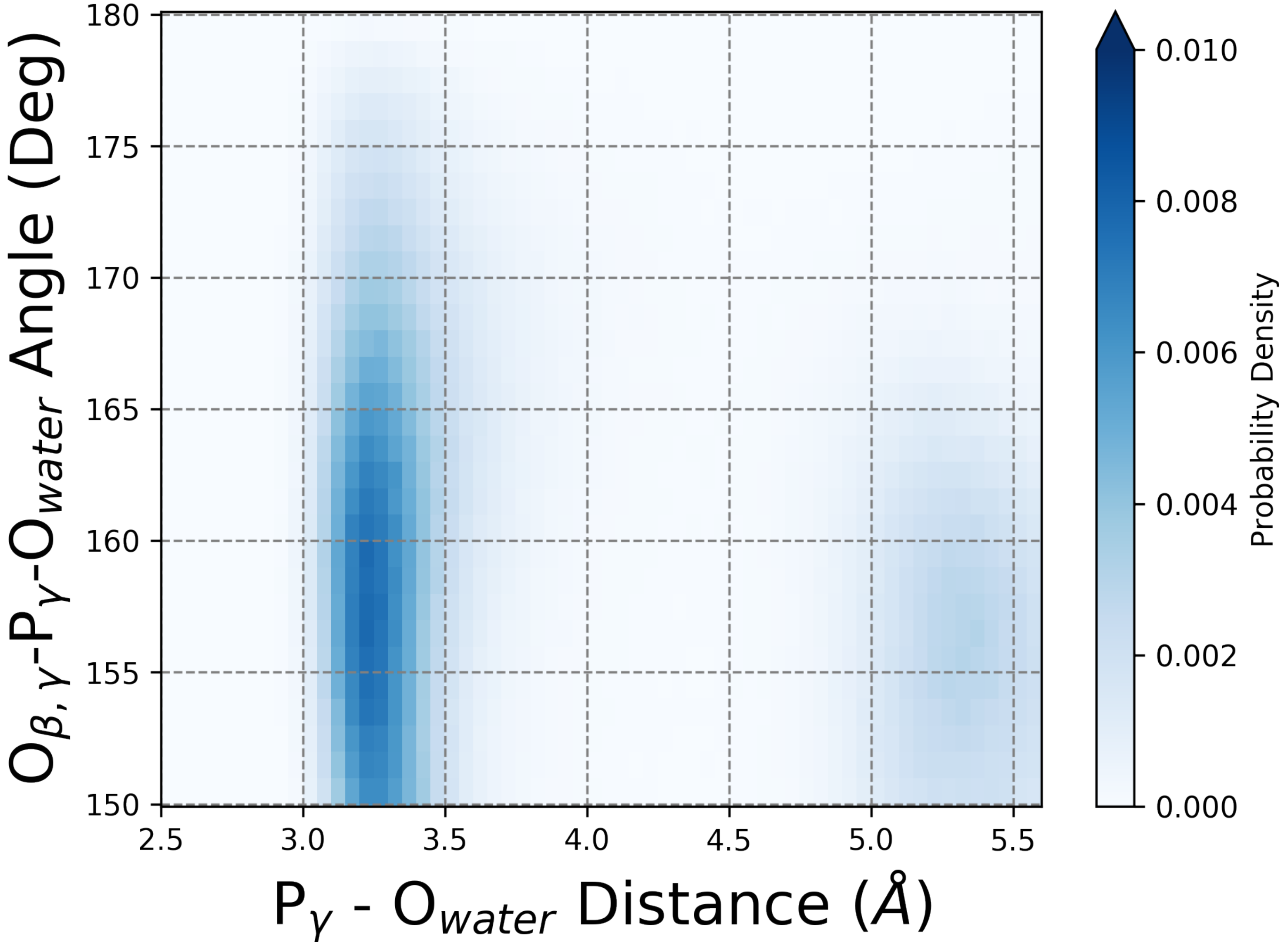

Figure G: Probability density heat map of water molecule positions within the NTPase active site for the ATP substrate state. The water positions are projected onto the P_γ-O_{wat} distance and $O_{\beta,\gamma}$-P_γ-O_{wat} angle. Waters within 5 Å and above an angle of 155° are defined as lytic waters.

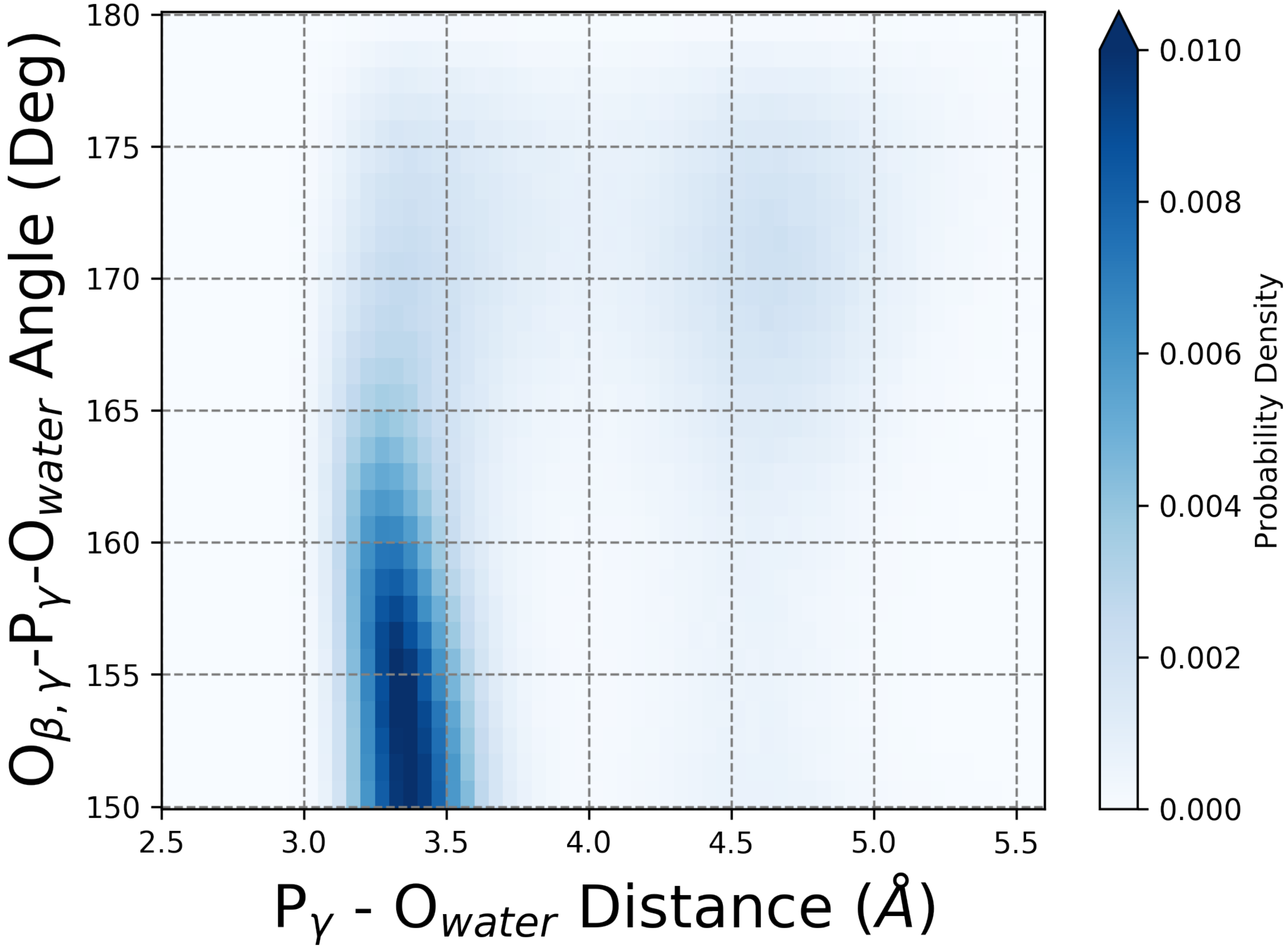

Figure H: Probability density heat map of water molecule positions within the NTPase active site for the ssRNA+ATP substrate state. The water positions are projected onto the P_γ-O_{wat} distance and $O_{\beta,\gamma}$-P_γ-O_{wat} angle. Waters within 5 Å and above an angle of 155° are defined as lytic waters.

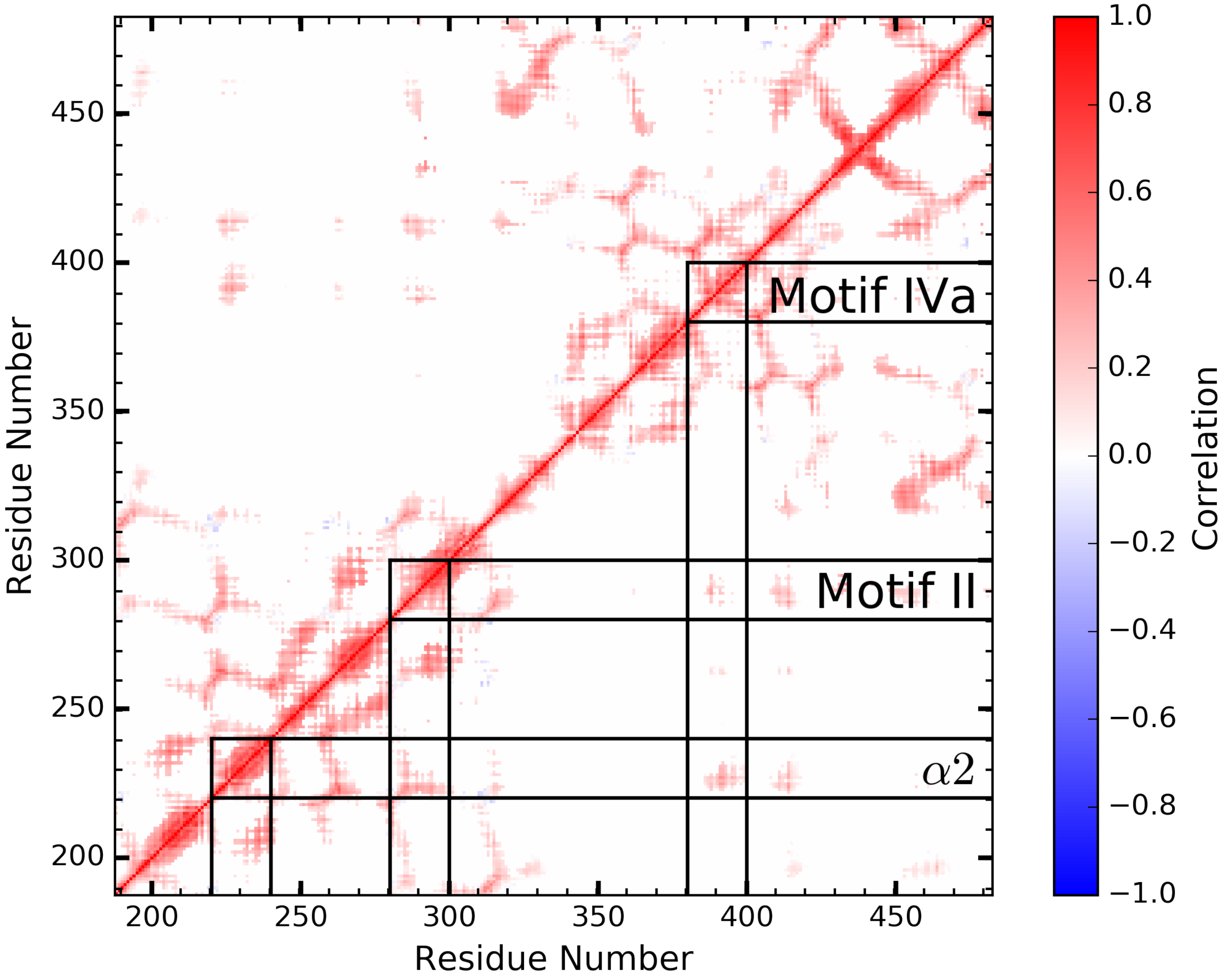

Figure I: Residue-residue correlation heat map for the Apo substrate state. Residues of $\alpha 2$, motif II, and motif IVa are highlighted by the drawn lines.

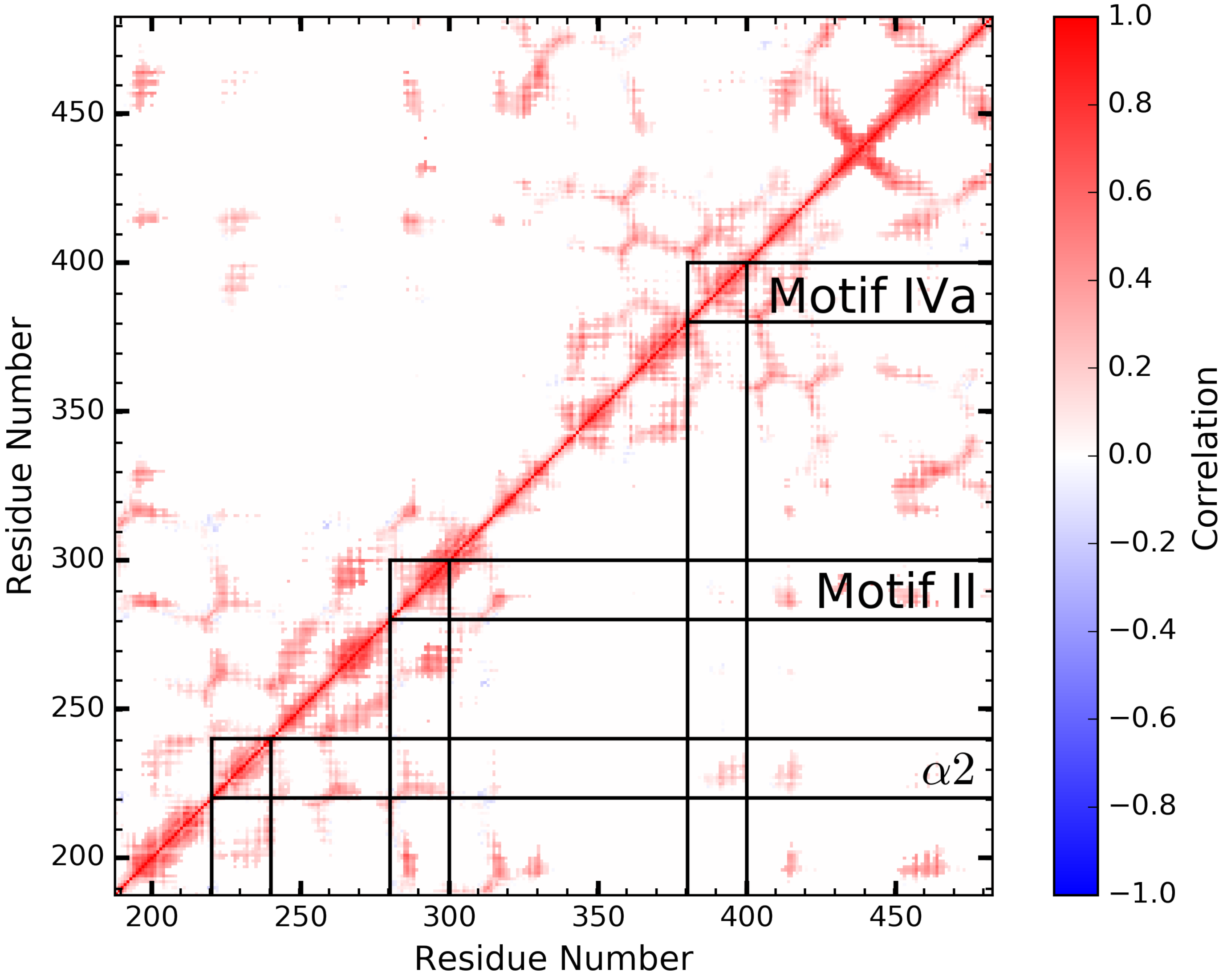

Figure J: Residue-residue correlation heat map for the ATP substrate state. Residues of $\alpha2$, motif II, and motif IVa are highlighted by the drawn lines.

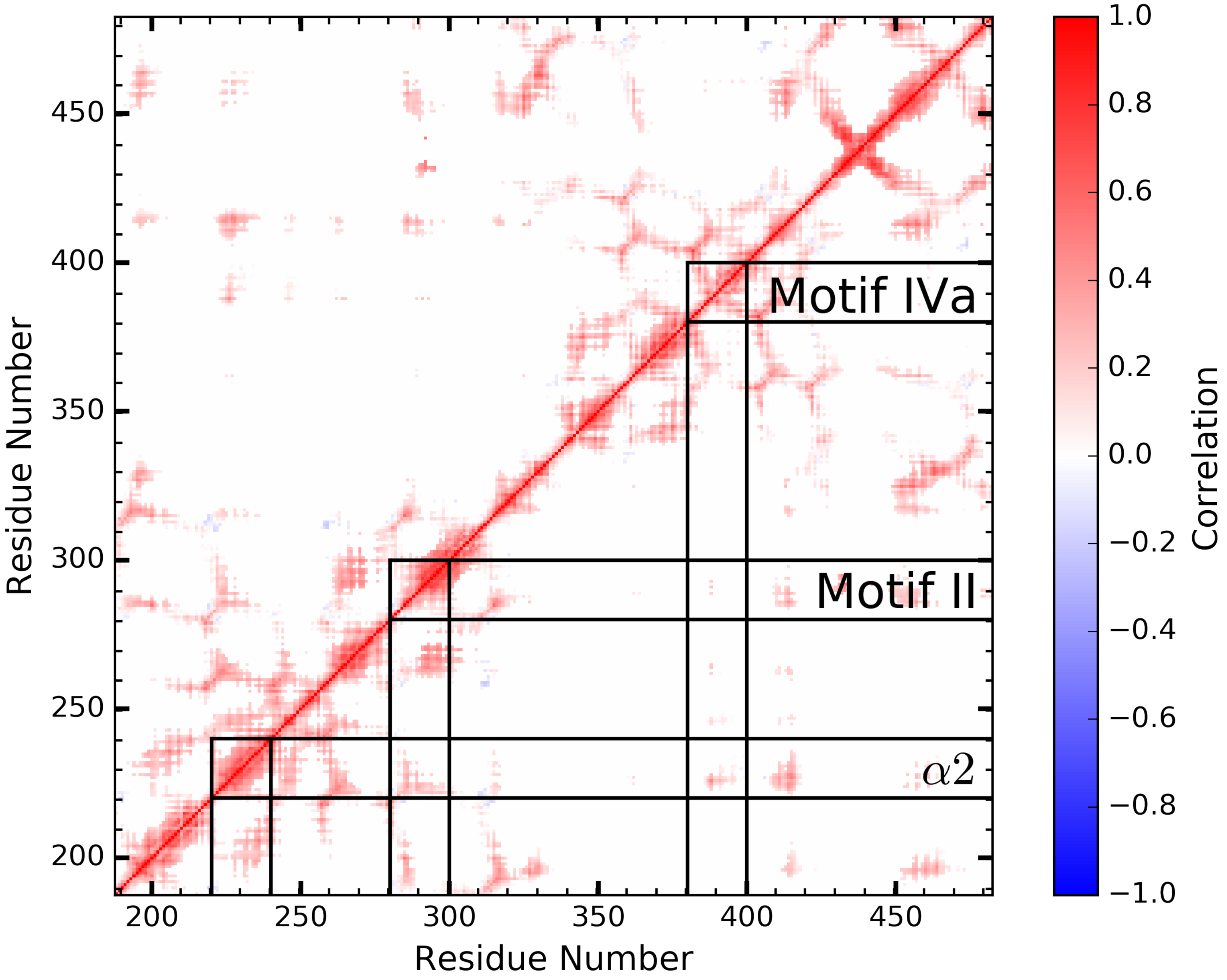

Figure K: Residue-residue correlation heat map for the ssRNA substrate state. Residues of $\alpha 2$, motif II, and motif IVa are highlighted by the drawn lines.

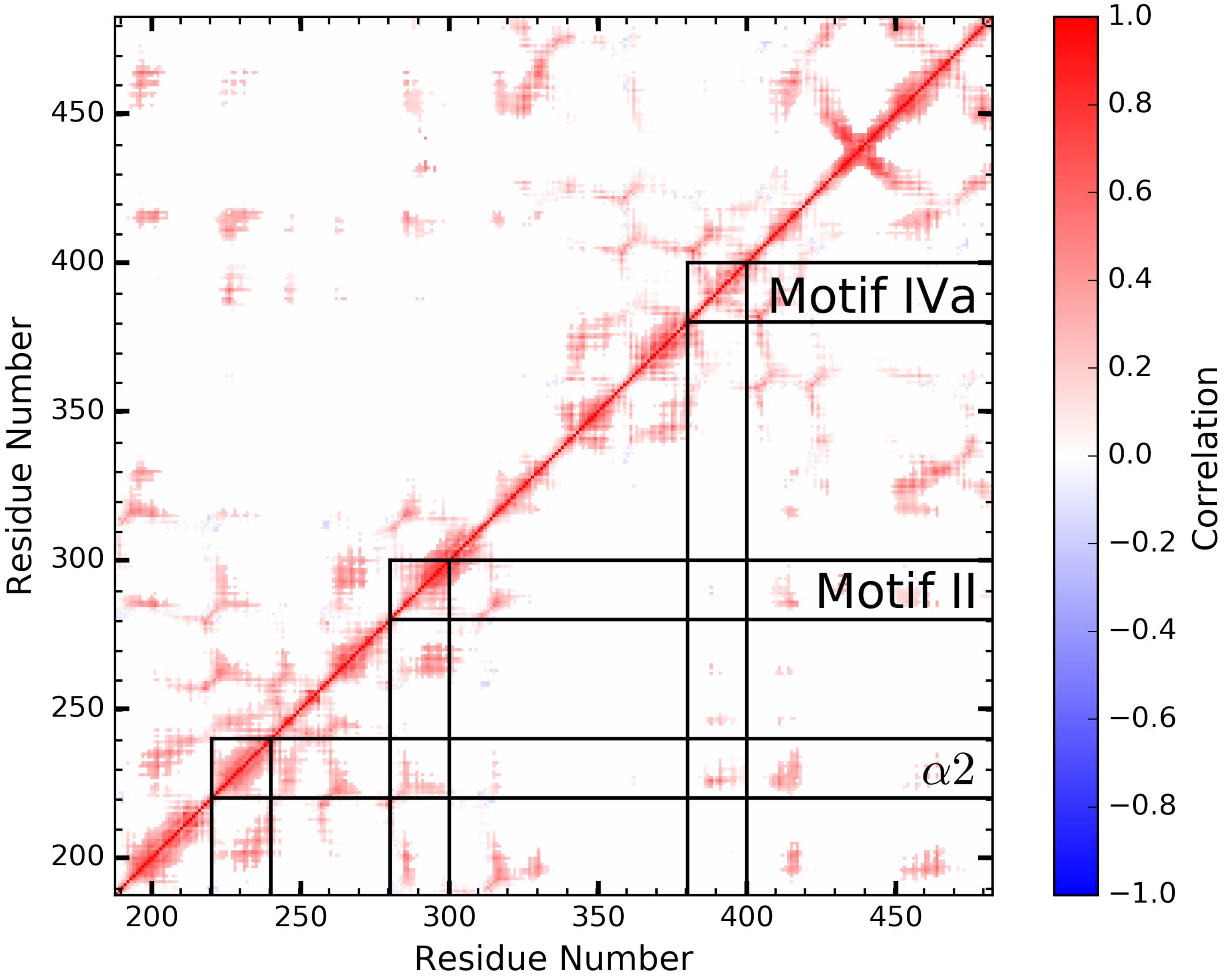

Figure L: Residue-residue correlation heat map for the ssRNA+ADP+P_i substrate state. Residues of $\alpha2$, motif II, and motif IVa are highlighted by the drawn lines.

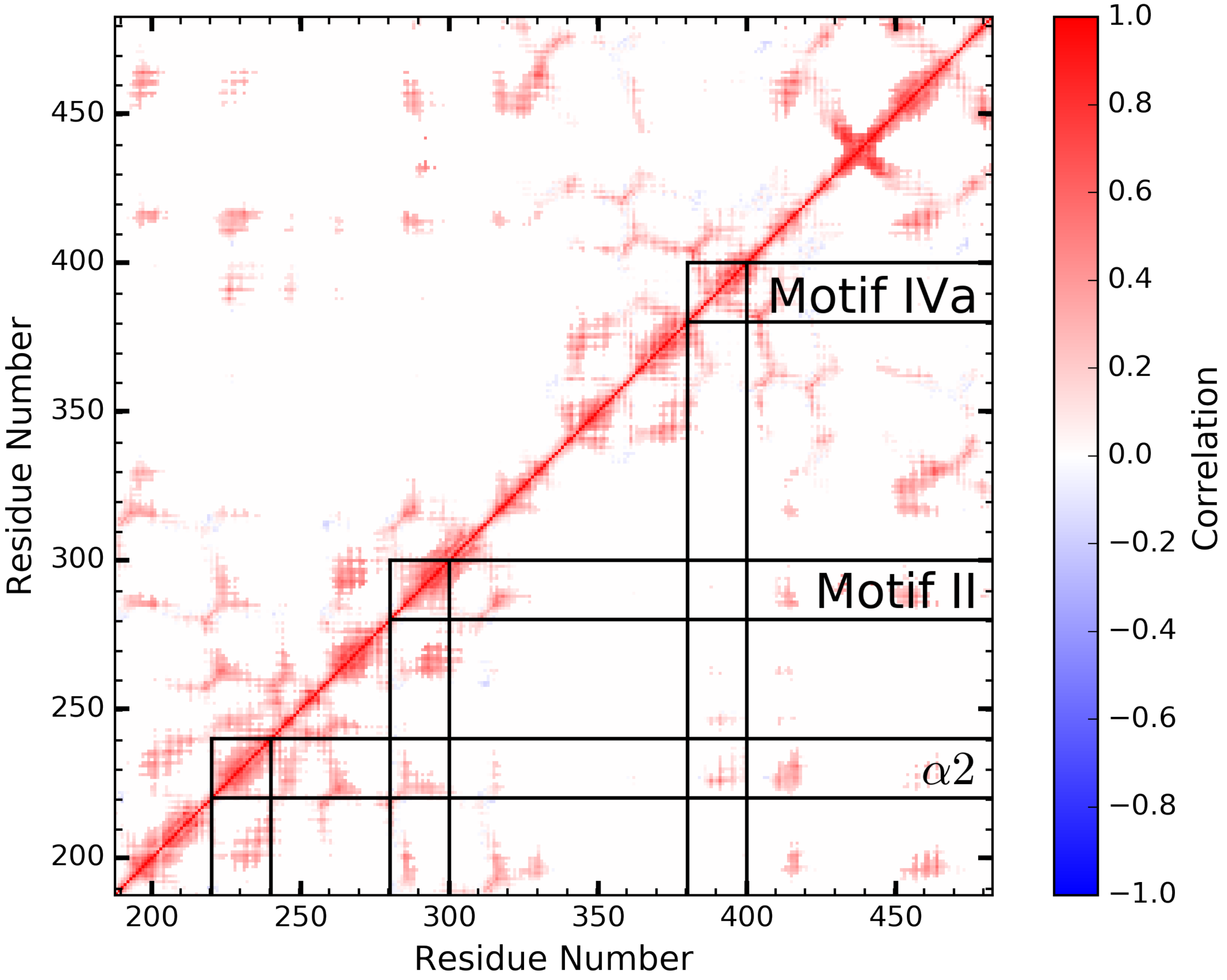

Figure M: Residue-residue correlation heat map for the ssRNA+ADP substrate state. Residues of $\alpha2$, motif II, and motif IVa are highlighted by the drawn lines.

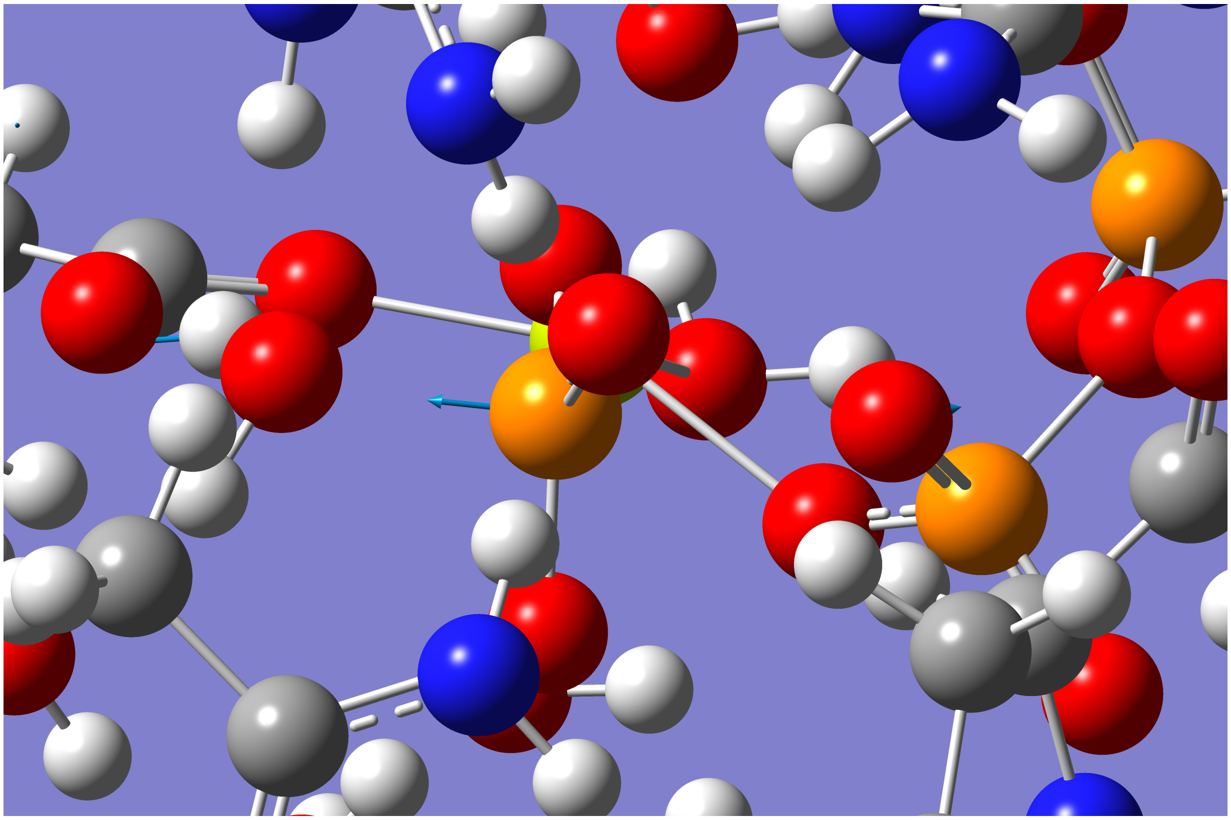

Figure N: Structural depiction of the imaginary frequency of the TS structure for the ATP substrate state. Vectors drawn show the magnitude of motion in the atoms in this frequency.

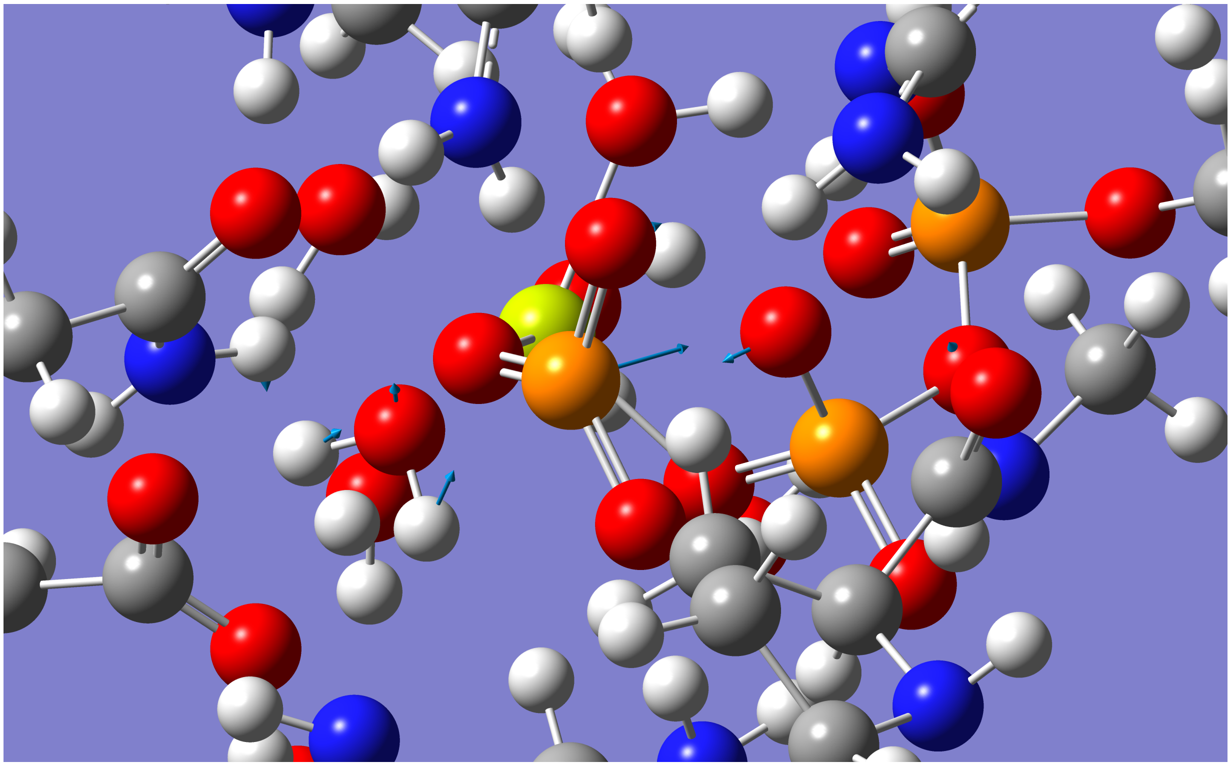

Figure O: Structural depiction of the imaginary frequency of the TS structure for the ssRNA+ATP substrate state. Vectors drawn show the magnitude of motion in the atoms in this frequency.

3 Coordinates of Optimized NTPase Active Site States - ssRNA+ATP Substrate State

3.1 ATP*

Atom	x	y	z
C	-0.285000	-0.600000	3.643000
N	0.620000	-0.629000	4.795000
C	1.980000	-0.961000	4.324000
C	1.925000	-0.859000	2.793000
C	0.486000	-1.259000	2.479000
C	-1.557000	-1.380000	3.930000
N	-2.690000	-1.032000	3.267000
C	-3.956000	-1.785000	3.315000
H	-4.400000	-1.738000	4.313000
O	-1.525000	-2.373000	4.664000
O	-3.645000	0.465000	0.708000
P	-4.764000	1.523000	1.312000
O	-6.088000	0.639000	1.199000
P	-7.018000	0.363000	-0.178000
O	-8.057000	-0.688000	0.470000
C	-7.622000	-1.982000	0.836000
H	-8.449000	-2.453000	1.370000
P	-2.100000	0.844000	0.219000
O	-1.357000	1.299000	1.473000
O	-1.585000	-0.483000	-0.326000
O	-2.227000	1.934000	-0.847000
Mg	-3.782000	3.101000	-1.377000
O	-2.902000	4.818000	-0.439000
O	-4.802000	2.700000	0.356000
O	-4.470000	1.776000	2.759000
O	-7.754000	1.597000	-0.557000
O	-6.097000	-0.335000	-1.157000
O	-5.370000	4.262000	-2.154000
H	-5.741000	3.651000	-2.806000
O	-2.656000	3.727000	-3.126000
O	-4.708000	1.554000	-2.533000
O	-0.461000	5.803000	-0.472000
C	0.713000	5.363000	-0.511000
O	1.080000	4.213000	-0.112000
C	1.792000	6.229000	-1.158000
C	2.306000	5.582000	-2.455000
H	2.737000	4.597000	-2.249000
N	-2.156000	3.050000	3.281000
C	-1.951000	4.432000	2.793000
H	-2.370000	5.143000	3.507000
O	-7.096000	4.224000	-0.144000
N	-2.248000	-2.908000	0.696000
C	-3.454000	-3.187000	0.208000
N	-4.237000	-2.203000	-0.207000
N	-3.865000	-4.472000	0.121000
C	-2.967000	-5.625000	0.123000

C	-2.772000	-6.279000	1.490000
H	-3.724000	-6.642000	1.890000
O	-0.214000	2.984000	-2.266000
O	1.102000	1.462000	0.303000
O	-3.474000	-0.402000	-4.278000
C	-2.259000	-0.406000	-4.290000
H	-1.712000	-1.273000	-4.720000
C	-1.426000	0.726000	-3.749000
N	0.013000	0.475000	-3.734000
H	0.408000	0.652000	-4.656000
N	2.871000	3.915000	2.539000
C	2.095000	3.964000	3.785000
H	2.800000	4.028000	4.623000
C	1.252000	2.705000	3.924000
O	4.005000	-0.630000	-1.080000
C	4.541000	0.323000	-0.487000
N	3.875000	1.119000	0.356000
C	6.004000	0.630000	-0.724000
H	6.580000	-0.297000	-0.671000
N	-0.579000	-2.312000	-2.050000
C	0.756000	-2.172000	-1.940000
N	1.260000	-1.233000	-1.159000
N	1.556000	-3.067000	-2.555000
C	1.053000	-4.189000	-3.343000
C	2.075000	-5.313000	-3.489000
H	2.364000	-5.709000	-2.511000
H	2.263000	-1.979000	4.628000
H	2.719000	-0.273000	4.752000
H	2.670000	-1.492000	2.300000
H	2.100000	0.171000	2.468000
H	0.380000	-2.352000	2.545000
H	0.134000	-0.926000	1.501000
H	-0.522000	0.427000	3.364000
H	-2.685000	-0.172000	2.733000
H	-4.637000	-1.332000	2.595000
H	-3.778000	-2.836000	3.064000
H	-2.454000	4.524000	1.828000
H	-0.884000	4.611000	2.654000
H	-3.161000	2.755000	3.280000
H	-1.737000	2.368000	2.590000
H	-1.745000	2.889000	4.200000
H	-6.092000	4.355000	-1.461000
H	3.076000	6.205000	-2.922000
H	1.492000	5.455000	-3.179000
H	2.627000	6.333000	-0.454000
H	1.384000	7.223000	-1.361000
H	2.245000	3.965000	1.731000
H	1.445000	4.854000	3.846000
H	0.628000	2.735000	4.824000
H	0.609000	2.577000	3.044000
H	1.886000	1.820000	3.997000
H	0.423000	1.178000	-3.121000
H	-1.770000	0.883000	-2.721000
H	-1.731000	1.633000	-4.296000

H	6.413000	1.353000	-0.013000
H	6.112000	1.032000	-1.736000
H	2.853000	1.066000	0.445000
H	4.315000	1.899000	0.824000
H	2.978000	-4.969000	-4.005000
H	1.642000	-6.125000	-4.079000
H	0.738000	-3.842000	-4.338000
H	0.169000	-4.583000	-2.830000
H	2.549000	-2.871000	-2.559000
H	-0.941000	-2.691000	-2.911000
H	-1.137000	-1.572000	-1.604000
H	2.275000	-1.067000	-1.075000
H	0.656000	-0.480000	-0.838000
H	-2.350000	-5.583000	2.224000
H	-2.090000	-7.131000	1.401000
H	-2.008000	-5.295000	-0.289000
H	-3.382000	-6.353000	-0.581000
H	-4.841000	-4.625000	-0.084000
H	-5.053000	-2.312000	-0.797000
H	-4.026000	-1.229000	0.024000
H	-1.783000	-3.533000	1.334000
H	-1.880000	-1.951000	0.559000
H	-6.745000	-1.938000	1.494000
H	-7.385000	-2.575000	-0.057000
H	-7.524000	3.352000	-0.312000
H	-6.378000	3.955000	0.454000
H	-2.846000	4.659000	-3.294000
H	-1.688000	3.670000	-2.920000
H	-0.790000	2.531000	-1.609000
H	0.400000	3.480000	-1.693000
H	-5.261000	0.881000	-2.055000
H	-4.251000	1.039000	-3.223000
H	0.336000	1.352000	0.903000
H	1.111000	2.424000	0.122000
H	-3.371000	5.658000	-0.508000
H	-1.926000	5.055000	-0.435000
H	3.471000	4.733000	2.476000
H	0.289000	-1.346000	5.433000

Table B: Cartesian coordinates of atoms in the optimized structure of the ATP* state.

3.2 TS

Atom	x	y	z
C	0.497000	0.135000	4.463000
N	0.134000	0.441000	5.864000
C	-1.327000	0.331000	5.944000
C	-1.848000	0.818000	4.587000
C	-0.813000	0.227000	3.624000
C	1.552000	1.135000	4.009000
N	2.791000	0.674000	3.766000
C	3.974000	1.478000	3.443000

H	4.778000	1.213000	4.134000
O	1.256000	2.345000	3.942000
O	3.310000	-0.637000	0.902000
P	4.212000	-1.673000	1.598000
O	5.757000	-1.135000	1.462000
P	6.622000	-0.812000	0.106000
O	7.966000	-0.243000	0.787000
C	7.887000	0.928000	1.586000
H	8.907000	1.159000	1.902000
P	1.007000	-1.153000	0.532000
O	0.950000	-1.519000	1.983000
O	0.850000	0.299000	0.113000
O	1.428000	-2.134000	-0.548000
Mg	3.318000	-2.782000	-1.042000
O	2.252000	-4.697000	-0.982000
O	4.099000	-3.000000	0.807000
O	3.996000	-1.844000	3.096000
O	6.955000	-2.085000	-0.620000
O	5.911000	0.289000	-0.664000
O	5.142000	-3.690000	-1.833000
H	5.805000	-2.975000	-1.655000
O	2.872000	-3.390000	-3.239000
O	4.015000	-1.032000	-1.941000
O	-0.291000	-4.891000	0.184000
C	-1.169000	-4.678000	-0.699000
O	-1.799000	-3.594000	-0.831000
C	-1.493000	-5.799000	-1.670000
C	-2.475000	-5.370000	-2.744000
H	-3.427000	-5.057000	-2.304000
N	1.921000	-3.440000	3.465000
C	1.748000	-4.628000	2.604000
H	2.407000	-5.431000	2.930000
O	6.237000	-4.704000	0.425000
N	1.912000	2.625000	1.157000
C	3.073000	2.831000	0.522000
N	3.679000	1.823000	-0.084000
N	3.584000	4.083000	0.462000
C	2.772000	5.297000	0.538000
C	2.980000	6.094000	1.819000
H	4.027000	6.400000	1.928000
O	1.504000	-6.890000	0.523000
O	-0.980000	-1.529000	0.375000
O	3.536000	0.417000	-4.342000
C	2.332000	0.521000	-4.238000
H	1.806000	1.400000	-4.674000
C	1.455000	-0.492000	-3.526000
N	0.021000	-0.299000	-3.753000
H	-0.291000	-0.827000	-4.562000
N	-1.896000	-3.459000	2.287000
C	-2.292000	-4.056000	3.568000
H	-3.231000	-3.579000	3.871000
C	-1.250000	-3.819000	4.654000
O	-4.198000	1.173000	-0.560000
C	-4.551000	-0.008000	-0.673000

N	-3.674000	-1.027000	-0.638000
C	-6.014000	-0.370000	-0.825000
H	-6.505000	-0.223000	0.142000
N	0.520000	2.340000	-1.745000
C	-0.815000	2.415000	-1.562000
N	-1.390000	1.659000	-0.642000
N	-1.534000	3.308000	-2.266000
C	-0.965000	4.290000	-3.182000
C	-1.830000	5.545000	-3.259000
H	-1.919000	6.017000	-2.276000
H	-1.699000	0.901000	6.800000
H	-1.600000	-0.722000	6.102000
H	-1.822000	1.914000	4.554000
H	-2.869000	0.487000	4.368000
H	-0.677000	0.826000	2.719000
H	-1.108000	-0.779000	3.312000
H	0.905000	-0.876000	4.411000
H	2.954000	-0.333000	3.690000
H	4.306000	1.268000	2.425000
H	3.729000	2.537000	3.557000
H	2.024000	-4.328000	1.595000
H	0.711000	-4.959000	2.619000
H	2.889000	-3.002000	3.392000
H	1.336000	-2.673000	3.060000
H	1.690000	-3.608000	4.442000
H	5.467000	-4.372000	-1.196000
H	-2.676000	-6.193000	-3.441000
H	-2.085000	-4.524000	-3.316000
H	-1.880000	-6.646000	-1.089000
H	-0.545000	-6.140000	-2.108000
H	-1.111000	-3.968000	1.875000
H	-2.494000	-5.135000	3.483000
H	-1.651000	-4.073000	5.640000
H	-0.369000	-4.450000	4.492000
H	-0.938000	-2.769000	4.664000
H	-0.494000	-0.655000	-2.953000
H	1.669000	-0.386000	-2.454000
H	1.825000	-1.494000	-3.778000
H	-6.170000	-1.403000	-1.146000
H	-6.478000	0.310000	-1.542000
H	-2.695000	-0.875000	-0.408000
H	-3.948000	-1.992000	-0.760000
H	-2.835000	5.314000	-3.629000
H	-1.378000	6.261000	-3.950000
H	-0.840000	3.850000	-4.180000
H	0.026000	4.564000	-2.806000
H	-2.537000	3.286000	-2.145000
H	0.863000	2.514000	-2.678000
H	0.970000	1.565000	-1.248000
H	-2.407000	1.635000	-0.505000
H	-0.787000	0.970000	-0.174000
H	2.706000	5.502000	2.697000
H	2.360000	6.997000	1.805000
H	1.727000	4.993000	0.438000

H	3.019000	5.913000	-0.335000
H	4.506000	4.167000	0.062000
H	4.573000	1.919000	-0.553000
H	3.512000	0.848000	0.257000
H	1.735000	3.063000	2.061000
H	1.534000	1.675000	1.072000
H	7.261000	0.751000	2.466000
H	7.484000	1.767000	1.006000
H	6.877000	-3.983000	0.282000
H	5.486000	-4.217000	0.825000
H	3.804000	-3.663000	-3.319000
H	2.408000	-4.195000	-2.959000
H	0.681000	-6.360000	0.409000
H	1.322000	-7.775000	0.190000
H	4.713000	-0.479000	-1.486000
H	4.100000	-0.846000	-2.889000
H	-1.212000	-1.820000	1.292000
H	-1.170000	-2.383000	-0.156000
H	2.446000	-5.539000	-0.527000
H	1.381000	-4.441000	-0.613000
H	-2.647000	-3.595000	1.611000
H	0.380000	1.412000	6.043000

Table C: Cartesian coordinates of atoms in the optimized structure of the Transition State.

3.3 HPO_4^{2-}

Atom	x	y	z
C	-0.099000	0.041000	3.477000
N	-0.981000	-0.363000	4.597000
C	-1.895000	0.747000	4.889000
C	-2.172000	1.324000	3.505000
C	-0.782000	1.274000	2.853000
C	1.300000	0.374000	3.987000
N	2.277000	-0.474000	3.621000
C	3.659000	-0.277000	4.020000
H	3.697000	0.069000	5.055000
O	1.491000	1.352000	4.725000
O	2.905000	-1.353000	1.081000
P	3.761000	-2.620000	1.158000
O	5.353000	-2.182000	1.138000
P	6.385000	-1.844000	-0.077000
O	7.758000	-1.732000	0.762000
C	7.874000	-0.732000	1.760000
H	8.868000	-0.840000	2.198000
P	0.309000	-1.040000	-0.452000
O	-0.200000	-1.860000	0.727000
O	0.739000	0.398000	-0.120000
O	1.158000	-1.778000	-1.480000
Mg	2.892000	-2.748000	-1.840000
O	1.478000	-4.369000	-2.236000
O	3.525000	-3.488000	-0.102000

O	3.575000	-3.373000	2.469000
O	6.509000	-2.984000	-1.044000
O	6.020000	-0.475000	-0.645000
O	4.577000	-3.825000	-2.761000
H	5.340000	-3.333000	-2.367000
O	2.548000	-2.734000	-4.138000
O	4.001000	-1.057000	-2.271000
O	-1.306000	-4.425000	-1.372000
C	-2.462000	-4.027000	-1.295000
O	-2.811000	-2.770000	-1.466000
C	-3.615000	-4.965000	-1.008000
C	-4.985000	-4.319000	-0.822000
H	-5.000000	-3.645000	0.039000
N	0.978000	-3.705000	2.351000
C	0.896000	-4.992000	1.625000
H	1.234000	-5.801000	2.273000
O	5.187000	-5.574000	-0.783000
N	2.339000	2.008000	1.558000
C	3.593000	1.865000	1.098000
N	3.830000	0.964000	0.154000
N	4.571000	2.685000	1.523000
C	4.402000	3.806000	2.438000
C	4.497000	3.419000	3.912000
H	5.450000	2.921000	4.119000
O	0.364000	-6.622000	-1.055000
O	-1.106000	-0.772000	-1.384000
O	3.200000	0.926000	-4.136000
C	2.075000	0.880000	-4.596000
H	1.790000	1.598000	-5.396000
C	0.976000	-0.049000	-4.166000
N	-0.260000	0.726000	-4.026000
H	-0.890000	0.571000	-4.806000
N	-3.049000	-1.497000	1.351000
C	-3.245000	-2.104000	2.672000
H	-2.574000	-1.602000	3.377000
C	-2.965000	-3.608000	2.694000
O	-3.342000	2.518000	-1.595000
C	-3.612000	1.473000	-0.995000
N	-2.968000	1.113000	0.141000
C	-4.649000	0.496000	-1.493000
H	-4.867000	0.701000	-2.542000
N	1.736000	2.481000	-1.817000
C	0.528000	3.026000	-1.980000
N	-0.466000	2.605000	-1.202000
N	0.330000	4.036000	-2.844000
C	1.320000	4.517000	-3.799000
C	0.755000	5.678000	-4.609000
H	0.468000	6.509000	-3.957000
H	-1.435000	1.512000	5.533000
H	-2.799000	0.370000	5.383000
H	-2.586000	2.336000	3.546000
H	-2.880000	0.683000	2.969000
H	-0.229000	2.180000	3.123000
H	-0.790000	1.182000	1.764000

H	-0.047000	-0.767000	2.741000
H	2.183000	-0.992000	2.740000
H	4.177000	-1.233000	3.917000
H	4.159000	0.463000	3.382000
H	1.561000	-4.922000	0.765000
H	-0.127000	-5.168000	1.290000
H	2.020000	-3.519000	2.563000
H	0.620000	-2.931000	1.735000
H	0.435000	-3.705000	3.213000
H	4.687000	-4.691000	-2.300000
H	-5.739000	-5.093000	-0.650000
H	-5.279000	-3.744000	-1.705000
H	-3.327000	-5.539000	-0.118000
H	-3.638000	-5.687000	-1.832000
H	-2.060000	-1.628000	1.097000
H	-4.273000	-1.909000	3.002000
H	-3.033000	-4.012000	3.712000
H	-3.678000	-4.158000	2.067000
H	-1.959000	-3.807000	2.306000
H	-0.740000	0.409000	-3.184000
H	1.240000	-0.529000	-3.220000
H	0.917000	-0.856000	-4.912000
H	-5.568000	0.618000	-0.910000
H	-4.314000	-0.540000	-1.378000
H	-2.415000	1.820000	0.607000
H	-3.199000	0.262000	0.693000
H	-0.120000	5.365000	-5.189000
H	1.511000	6.040000	-5.312000
H	1.619000	3.704000	-4.472000
H	2.209000	4.848000	-3.250000
H	-0.633000	4.300000	-3.001000
H	2.495000	2.668000	-2.453000
H	1.789000	1.620000	-1.261000
H	-1.441000	2.805000	-1.434000
H	-0.252000	1.741000	-0.678000
H	3.688000	2.746000	4.218000
H	4.439000	4.318000	4.535000
H	3.439000	4.279000	2.217000
H	5.175000	4.537000	2.185000
H	5.510000	2.430000	1.252000
H	4.801000	0.734000	-0.099000
H	3.295000	0.077000	0.271000
H	2.188000	2.334000	2.505000
H	1.660000	1.333000	1.185000
H	7.114000	-0.869000	2.538000
H	7.778000	0.268000	1.317000
H	5.989000	-5.030000	-0.703000
H	4.536000	-5.015000	-0.311000
H	3.444000	-3.106000	-4.228000
H	1.969000	-3.512000	-4.108000
H	-0.429000	-6.064000	-1.166000
H	0.240000	-7.403000	-1.606000
H	4.815000	-0.770000	-1.779000
H	3.752000	-0.363000	-2.906000

H	-1.671000	-0.158000	-0.879000
H	-2.024000	-2.146000	-1.521000
H	1.448000	-5.254000	-1.821000
H	0.658000	-3.936000	-1.932000
H	-3.571000	-2.026000	0.652000
H	-0.442000	-0.619000	5.418000

Table D: Cartesian coordinates of atoms in the optimized structure of the HPO_4^{2-} product state.

4 Coordinates of Optimized NTPase Active Site States - ATP Substrate State

4.1 ATP*

Atom	x	y	z
N	2.583000	4.626000	0.782000
C	2.958000	4.976000	-0.594000
H	2.948000	6.062000	-0.727000
H	2.211000	4.542000	-1.264000
C	4.326000	4.322000	-0.866000
H	5.137000	5.015000	-0.613000
H	4.432000	4.039000	-1.917000
C	4.344000	3.096000	0.075000
H	5.217000	3.092000	0.735000
H	4.349000	2.155000	-0.480000
C	3.040000	3.234000	0.900000
H	2.262000	2.615000	0.433000
C	3.141000	2.728000	2.326000
O	3.930000	1.821000	2.635000
N	2.255000	3.257000	3.208000
H	1.511000	3.843000	2.817000
C	2.024000	2.788000	4.586000
H	1.144000	2.143000	4.632000
H	2.917000	2.263000	4.925000
H	1.850000	3.655000	5.227000
H	3.126000	5.204000	1.425000
H	-1.981000	6.491000	-0.639000
C	-1.985000	5.403000	-0.556000
H	-2.531000	5.082000	0.332000
H	-2.442000	4.962000	-1.442000
N	-0.586000	4.913000	-0.469000
H	-0.091000	5.051000	-1.393000
H	-0.067000	5.310000	0.320000
H	-0.545000	3.886000	-0.298000
H	-4.769000	2.262000	3.028000
O	-5.185000	1.863000	2.222000
H	-5.661000	1.093000	2.560000
H	-6.051000	0.188000	-3.204000
C	-5.650000	-0.826000	-3.319000
H	-6.407000	-1.424000	-3.840000
H	-4.760000	-0.766000	-3.950000

C	-5.302000	-1.441000	-1.968000
H	-6.181000	-1.525000	-1.321000
H	-4.879000	-2.441000	-2.112000
C	-4.241000	-0.604000	-1.282000
O	-3.241000	-0.284000	-1.974000
O	-4.445000	-0.252000	-0.080000
N	0.408000	4.956000	-3.161000
H	1.308000	4.476000	-3.237000
H	-0.231000	4.339000	-3.672000
C	0.473000	6.255000	-3.846000
H	-0.535000	6.689000	-3.834000
C	1.445000	7.195000	-3.145000
H	1.477000	8.170000	-3.645000
H	1.151000	7.355000	-2.101000
H	2.459000	6.779000	-3.149000
H	0.763000	6.146000	-4.901000
H	-2.617000	-4.434000	-1.218000
N	-3.036000	-3.607000	-0.802000
H	-2.313000	-3.093000	-0.307000
C	-4.141000	-3.954000	0.093000
H	-4.503000	-3.021000	0.547000
H	-4.976000	-4.363000	-0.488000
C	-3.857000	-4.926000	1.240000
O	-4.651000	-5.737000	1.657000
H	-2.842000	-4.840000	1.689000
H	2.047000	-0.644000	-4.193000
C	2.192000	0.394000	-4.507000
H	3.164000	0.507000	-4.994000
H	1.415000	0.646000	-5.237000
C	2.084000	1.367000	-3.355000
O	2.606000	2.483000	-3.386000
N	1.385000	0.917000	-2.292000
H	1.086000	1.601000	-1.599000
H	0.781000	0.100000	-2.364000
H	4.435000	-5.910000	0.384000
C	3.525000	-6.204000	-0.147000
H	3.765000	-6.354000	-1.207000
H	3.184000	-7.162000	0.254000
C	2.424000	-5.157000	0.030000
H	1.508000	-5.489000	-0.478000
H	2.194000	-5.036000	1.094000
N	2.842000	-3.855000	-0.489000
H	3.549000	-3.845000	-1.210000
C	2.227000	-2.687000	-0.213000
N	1.125000	-2.654000	0.536000
H	0.465000	-3.418000	0.521000
H	0.714000	-1.721000	0.662000
N	2.737000	-1.535000	-0.644000
H	3.678000	-1.469000	-0.999000
H	2.212000	-0.671000	-0.470000
H	4.075000	-0.920000	4.268000
C	3.677000	-1.405000	5.158000
H	3.198000	-2.343000	4.861000
H	4.533000	-1.650000	5.798000

C	2.714000	-0.532000	5.956000
H	2.627000	-0.920000	6.974000
H	3.098000	0.491000	6.035000
N	1.355000	-0.523000	5.424000
H	0.590000	-0.542000	6.084000
C	1.020000	-0.394000	4.136000
N	-0.275000	-0.587000	3.779000
H	-0.990000	-0.447000	4.512000
H	-0.525000	0.058000	3.030000
N	1.918000	-0.146000	3.197000
H	2.779000	0.363000	3.390000
H	1.576000	-0.024000	2.229000
MG	-3.527000	1.156000	1.072000
O	-0.137000	2.242000	-0.354000
P	-0.278000	1.110000	0.657000
O	0.893000	0.120000	0.679000
O	-1.620000	0.359000	0.538000
O	-0.237000	1.760000	2.165000
P	-1.247000	2.995000	2.632000
O	-2.538000	2.763000	1.891000
O	-0.501000	4.281000	2.500000
O	-1.384000	2.601000	4.195000
P	-2.651000	1.921000	5.037000
O	-2.468000	0.417000	4.998000
O	-3.942000	2.525000	4.585000
O	-2.222000	2.398000	6.513000
C	-2.351000	3.782000	6.836000
H	-1.674000	4.383000	6.220000
H	-2.080000	3.880000	7.889000
H	-3.382000	4.117000	6.683000
O	-1.634000	-2.166000	1.608000
H	-1.115000	-2.024000	2.418000
H	-1.614000	-1.287000	1.160000
O	-0.685000	-1.292000	-1.722000
H	-1.468000	-1.389000	-2.290000
H	-1.074000	-0.753000	-1.005000
O	-1.104000	2.819000	-4.646000
H	-1.823000	2.883000	-5.285000
H	-1.509000	2.458000	-3.824000
O	-2.114000	2.077000	-2.229000
H	-2.446000	1.142000	-2.155000
H	-1.353000	2.115000	-1.607000
O	-4.069000	2.599000	-0.454000
H	-3.418000	2.538000	-1.195000
H	-4.925000	2.348000	-0.822000
O	-3.600000	-0.343000	2.645000
H	-3.273000	-0.144000	3.556000
H	-3.214000	-1.196000	2.383000

Table E: Cartesian coordinates of atoms in the optimized structure of the ATP* state.

4.2 TS

Atom	x	y	z
N	2.590000	4.634000	0.803000
C	3.039000	5.067000	-0.520000
H	3.073000	6.159000	-0.570000
H	2.298000	4.737000	-1.261000
C	4.407000	4.398000	-0.768000
H	5.211000	5.062000	-0.434000
H	4.582000	4.185000	-1.828000
C	4.364000	3.119000	0.099000
H	5.220000	3.057000	0.777000
H	4.369000	2.210000	-0.511000
C	3.042000	3.235000	0.899000
H	2.263000	2.611000	0.440000
C	3.141000	2.728000	2.326000
O	3.993000	1.880000	2.651000
N	2.237000	3.231000	3.194000
H	1.449000	3.738000	2.777000
C	2.024000	2.788000	4.586000
H	1.363000	1.921000	4.619000
H	2.987000	2.585000	5.056000
H	1.536000	3.610000	5.116000
H	3.068000	5.187000	1.512000
H	-1.775000	6.473000	-0.525000
C	-1.985000	5.403000	-0.556000
H	-2.568000	5.117000	0.320000
H	-2.515000	5.144000	-1.473000
N	-0.699000	4.660000	-0.526000
H	-0.091000	4.927000	-1.338000
H	-0.233000	4.728000	0.401000
H	-0.821000	3.641000	-0.657000
H	-4.243000	1.373000	2.478000
O	-4.614000	1.035000	1.609000
H	-5.200000	0.306000	1.849000
H	-6.014000	-0.100000	-2.584000
C	-5.651000	-0.826000	-3.319000
H	-6.519000	-1.300000	-3.788000
H	-5.108000	-0.279000	-4.099000
C	-4.758000	-1.869000	-2.651000
H	-5.325000	-2.393000	-1.877000
H	-4.398000	-2.599000	-3.382000
C	-3.576000	-1.188000	-2.002000
O	-2.500000	-1.142000	-2.676000
O	-3.770000	-0.633000	-0.891000
N	0.301000	4.947000	-3.199000
H	1.011000	4.306000	-3.563000
H	-0.585000	4.528000	-3.486000
C	0.473000	6.255000	-3.846000
H	-0.399000	6.873000	-3.597000
C	1.742000	6.939000	-3.356000
H	1.895000	7.894000	-3.872000
H	1.688000	7.135000	-2.280000
H	2.619000	6.309000	-3.545000

H	0.502000	6.166000	-4.941000
H	-2.913000	-4.408000	-1.417000
N	-3.036000	-3.608000	-0.802000
H	-2.154000	-3.451000	-0.324000
C	-4.106000	-3.865000	0.165000
H	-4.313000	-2.923000	0.694000
H	-5.027000	-4.135000	-0.361000
C	-3.857000	-4.926000	1.240000
O	-4.721000	-5.617000	1.725000
H	-2.798000	-5.026000	1.572000
H	1.226000	0.286000	-5.008000
C	2.192000	0.394000	-4.507000
H	2.294000	-0.359000	-3.720000
H	2.977000	0.236000	-5.252000
C	2.312000	1.815000	-3.985000
O	2.160000	2.789000	-4.718000
N	2.655000	1.958000	-2.674000
H	2.569000	2.903000	-2.320000
H	2.376000	1.256000	-1.991000
H	4.045000	-6.025000	0.802000
C	3.525000	-6.204000	-0.146000
H	4.264000	-6.239000	-0.953000
H	3.046000	-7.184000	-0.078000
C	2.474000	-5.134000	-0.412000
H	1.961000	-5.325000	-1.360000
H	1.721000	-5.127000	0.385000
N	3.114000	-3.824000	-0.510000
H	3.992000	-3.697000	-0.027000
C	2.563000	-2.744000	-1.079000
N	1.293000	-2.783000	-1.488000
H	0.618000	-3.250000	-0.880000
H	0.913000	-1.934000	-1.917000
N	3.287000	-1.640000	-1.306000
H	4.266000	-1.629000	-1.061000
H	2.752000	-0.765000	-1.177000
H	3.929000	-1.805000	4.170000
C	3.678000	-1.405000	5.158000
H	3.189000	-2.199000	5.734000
H	4.615000	-1.143000	5.661000
C	2.798000	-0.187000	5.034000
H	2.540000	0.218000	6.015000
H	3.325000	0.597000	4.488000
N	1.542000	-0.484000	4.360000
H	0.697000	-0.569000	4.913000
C	1.384000	-0.573000	3.042000
N	0.178000	-0.959000	2.571000
H	-0.578000	-0.862000	3.271000
H	-0.080000	-0.409000	1.743000
N	2.389000	-0.380000	2.181000
H	3.148000	0.242000	2.473000
H	2.079000	-0.167000	1.223000
MG	-2.876000	0.432000	0.526000
O	-0.289000	1.997000	-1.312000
P	0.067000	0.650000	-0.734000

O	1.530000	0.251000	-0.567000
O	-0.982000	-0.211000	0.000000
O	0.326000	1.625000	1.340000
P	-0.794000	2.460000	1.978000
O	-2.148000	2.167000	1.284000
O	-0.466000	3.930000	2.144000
O	-0.938000	1.840000	3.515000
P	-2.141000	1.118000	4.335000
O	-1.905000	-0.388000	4.254000
O	-3.493000	1.644000	3.944000
O	-1.734000	1.545000	5.837000
C	-1.868000	2.922000	6.185000
H	-1.254000	3.546000	5.525000
H	-1.519000	3.016000	7.216000
H	-2.913000	3.235000	6.115000
O	-0.618000	-2.797000	0.630000
H	-0.134000	-2.589000	1.451000
H	-0.739000	-1.891000	0.245000
O	-0.223000	-0.362000	-2.305000
H	-1.257000	-0.713000	-2.296000
H	-0.253000	0.181000	-3.139000
O	-1.214000	0.680000	-4.556000
H	-1.913000	0.028000	-4.386000
H	-1.606000	1.533000	-4.264000
O	-2.079000	2.885000	-3.093000
H	-2.900000	2.756000	-2.587000
H	-1.403000	2.516000	-2.479000
O	-3.686000	2.393000	-0.786000
H	-3.162000	2.743000	-0.031000
H	-4.592000	2.337000	-0.452000
O	-2.910000	-1.210000	1.912000
H	-2.680000	-0.981000	2.854000
H	-2.315000	-1.933000	1.656000

Table F: Cartesian coordinates of atoms in the optimized structure of the Transition State.

4.3 HPO_4^{2-}

Atom	x	y	z
N	2.571000	4.608000	0.793000
C	2.978000	5.015000	-0.550000
H	3.051000	6.106000	-0.606000
H	2.196000	4.711000	-1.258000
C	4.305000	4.291000	-0.859000
H	5.152000	4.927000	-0.580000
H	4.408000	4.053000	-1.923000
C	4.260000	3.028000	0.031000
H	5.144000	2.948000	0.671000
H	4.213000	2.108000	-0.560000
C	2.984000	3.198000	0.891000
H	2.164000	2.584000	0.487000
C	3.141000	2.727000	2.327000

O	4.004000	1.885000	2.643000
N	2.274000	3.267000	3.209000
H	1.480000	3.763000	2.791000
C	2.025000	2.797000	4.587000
H	1.369000	1.926000	4.574000
H	2.974000	2.583000	5.080000
H	1.514000	3.603000	5.117000
H	3.088000	5.154000	1.478000
H	-1.746000	6.470000	-0.526000
C	-1.988000	5.407000	-0.560000
H	-2.655000	5.159000	0.267000
H	-2.451000	5.149000	-1.513000
N	-0.735000	4.626000	-0.401000
H	-0.048000	4.919000	-1.117000
H	-0.381000	4.638000	0.589000
H	-0.854000	3.616000	-0.598000
H	-4.274000	1.142000	2.651000
O	-4.602000	0.876000	1.736000
H	-5.133000	0.083000	1.882000
H	-6.078000	-0.064000	-2.655000
C	-5.645000	-0.823000	-3.315000
H	-6.462000	-1.288000	-3.876000
H	-4.984000	-0.323000	-4.029000
C	-4.890000	-1.878000	-2.509000
H	-5.561000	-2.362000	-1.798000
H	-4.458000	-2.634000	-3.169000
C	-3.790000	-1.220000	-1.738000
O	-2.656000	-1.117000	-2.383000
O	-3.983000	-0.706000	-0.631000
N	0.121000	5.011000	-3.146000
H	0.576000	4.228000	-3.624000
H	-0.872000	4.812000	-3.267000
C	0.473000	6.253000	-3.845000
H	-0.021000	7.089000	-3.335000
C	1.981000	6.465000	-3.841000
H	2.248000	7.370000	-4.396000
H	2.360000	6.564000	-2.820000
H	2.489000	5.616000	-4.314000
H	0.114000	6.254000	-4.886000
H	-2.939000	-4.421000	-1.417000
N	-3.039000	-3.616000	-0.805000
H	-2.142000	-3.458000	-0.350000
C	-4.094000	-3.848000	0.185000
H	-4.250000	-2.908000	0.733000
H	-5.037000	-4.082000	-0.319000
C	-3.857000	-4.925000	1.241000
O	-4.726000	-5.624000	1.704000
H	-2.801000	-5.031000	1.581000
H	2.540000	-0.276000	-3.717000
C	2.192000	0.396000	-4.505000
H	2.961000	0.459000	-5.283000
H	1.290000	-0.013000	-4.960000
C	1.921000	1.793000	-4.008000
O	1.295000	2.604000	-4.700000

N	2.445000	2.138000	-2.808000
H	2.049000	2.978000	-2.404000
H	2.561000	1.406000	-2.114000
H	3.880000	-6.018000	0.873000
C	3.526000	-6.205000	-0.148000
H	4.383000	-6.195000	-0.828000
H	3.083000	-7.204000	-0.166000
C	2.479000	-5.173000	-0.569000
H	2.127000	-5.377000	-1.585000
H	1.613000	-5.216000	0.102000
N	3.051000	-3.826000	-0.558000
H	3.839000	-3.666000	0.054000
C	2.527000	-2.750000	-1.163000
N	1.324000	-2.823000	-1.748000
H	0.604000	-3.318000	-1.228000
H	0.971000	-1.964000	-2.185000
N	3.215000	-1.605000	-1.249000
H	4.141000	-1.567000	-0.847000
H	2.614000	-0.757000	-1.123000
H	4.001000	-1.820000	4.195000
C	3.677000	-1.410000	5.157000
H	3.167000	-2.205000	5.714000
H	4.572000	-1.120000	5.717000
C	2.781000	-0.213000	4.955000
H	2.453000	0.198000	5.912000
H	3.322000	0.576000	4.430000
N	1.577000	-0.550000	4.210000
H	0.688000	-0.607000	4.701000
C	1.492000	-0.628000	2.888000
N	0.307000	-1.019000	2.345000
H	-0.437000	-0.908000	3.048000
H	0.084000	-0.321000	1.618000
N	2.520000	-0.411000	2.061000
H	3.248000	0.234000	2.382000
H	2.193000	-0.169000	1.115000
MG	-2.803000	0.471000	0.642000
O	-0.247000	2.058000	-1.403000
P	0.066000	0.598000	-1.134000
O	1.450000	0.263000	-0.561000
O	-1.105000	-0.147000	-0.385000
O	0.273000	1.574000	1.832000
P	-0.845000	2.511000	2.232000
O	-2.157000	2.183000	1.454000
O	-0.492000	3.990000	2.237000
O	-1.206000	2.167000	3.833000
P	-2.163000	1.040000	4.480000
O	-1.612000	-0.358000	4.197000
O	-3.608000	1.283000	4.134000
O	-1.876000	1.300000	6.047000
C	-2.323000	2.528000	6.614000
H	-1.804000	3.375000	6.150000
H	-2.084000	2.484000	7.678000
H	-3.404000	2.642000	6.480000
O	-0.583000	-2.707000	0.399000

H	-0.032000	-2.466000	1.173000
H	-0.731000	-1.815000	-0.007000
O	0.087000	-0.211000	-2.567000
H	-1.980000	-0.726000	-1.769000
H	-0.418000	0.229000	-3.305000
O	-1.142000	1.079000	-4.608000
H	-0.442000	1.686000	-4.912000
H	-1.792000	1.671000	-4.175000
O	-2.300000	2.927000	-2.906000
H	-3.049000	2.735000	-2.317000
H	-1.528000	2.591000	-2.389000
O	-3.734000	2.271000	-0.559000
H	-3.213000	2.702000	0.161000
H	-4.625000	2.184000	-0.188000
O	-2.703000	-1.228000	1.913000
H	-2.445000	-1.004000	2.851000
H	-2.089000	-1.918000	1.608000

Table G: Cartesian coordinates of atoms in the optimized structure of the HPO_4^{2-} product state.

References

[1] Roe, D. R. and Cheatham, T. E. (2013) PTRAJ and CPPTRAJ: Software for Processing and Analysis of Molecular Dynamics Trajectory Data. *J. Chem. Theory Comput.,* **9**(7), 3084–3095.

[2] Van Wart, A. T., Eargle, J., Luthey-Schulten, Z., and Amaro, R. E. (2012) Exploring Residue Component Contributions to Dynamical Network Models of Allostery. *J. Chem. Theory Comput.,* **8**(8), 2949–2961.

[3] Seo, M.-H., Park, J., Kim, E., Hohng, S., and Kim, H.-S. (2014) Protein conformational dynamics dictate the binding affinity for a ligand. *Nat. Commun.,* **5**, 3724.

[4] Van Wart, A. T., Durrant, J., Votapka, L., and Amaro, R. E. (2014) Weighted Implementation of Suboptimal Paths (WISP): An Optimized Algorithm and Tool for Dynamical Network Analysis. *J. Chem. Theory Comput.,* **10**(2), 511–517.

[5] Doshi, U., Holliday, M. J., Eisenmesser, E. Z., and Hamelberg, D. (2016) Dynamical network of residue-residue contacts reveals coupled allosteric effects in recognition, catalysis, and mutation. *Proc. Natl. Acad. Sci. U. S. A.,* **113**(17), 4735–4740.
